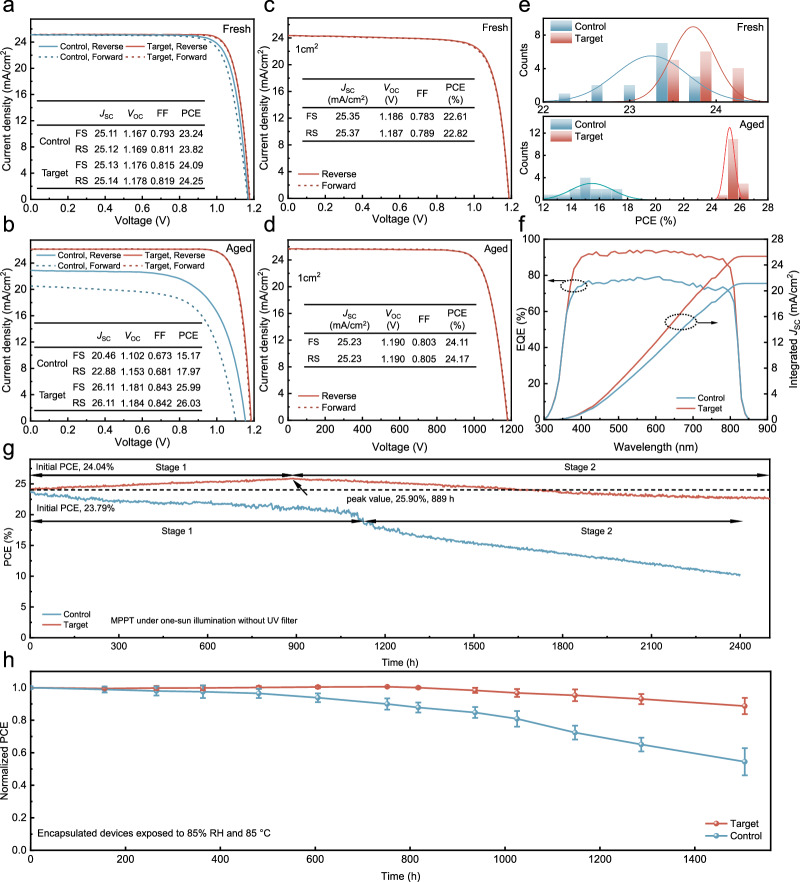# Author Correction: Silver coordination-induced n-doping of PCBM for stable and efficient inverted perovskite solar cells

**DOI:** 10.1038/s41467-024-49694-z

**Published:** 2024-06-25

**Authors:** Cheng Gong, Haiyun Li, Huaxin Wang, Cong Zhang, Qixin Zhuang, Awen Wang, Zhiyuan Xu, Wensi Cai, Ru Li, Xiong Li, Zhigang Zang

**Affiliations:** 1 Key Laboratory of Optoelectronic Technology & Systems (Ministry of Education), Chongqing University, Chongqing, 400044 China; 2 Wuhan National Laboratory for Optoelectronics, Huazhong University of Science and Technology, Wuhan, 430074 Hubei China; 3 College of Information Science and Engineering, Yanshan University, Qinhuangdao, 066004 China

**Keywords:** Solar cells, Optical materials, Solar cells

Correction to: *Nature Communications* 10.1038/s41467-024-49395-7, published online 10 June 2024

The original version of this article contained an error in Fig. 3, in which the *J*_*SC*_ parameter in the insets of Fig. 3c was incorrect. The correct values should be 24.34 for the FS condition and 24.36 for the RS condition. There is a unit error in the horizontal axis of the JV curve in Fig. 3d. The unit should be “Voltage (mV)”; however, same units for Fig. 3c, 3d are preferable and therefore the values on the horizontal axis have been corrected. The original article has been corrected.

The correct version of Fig. 3 is:
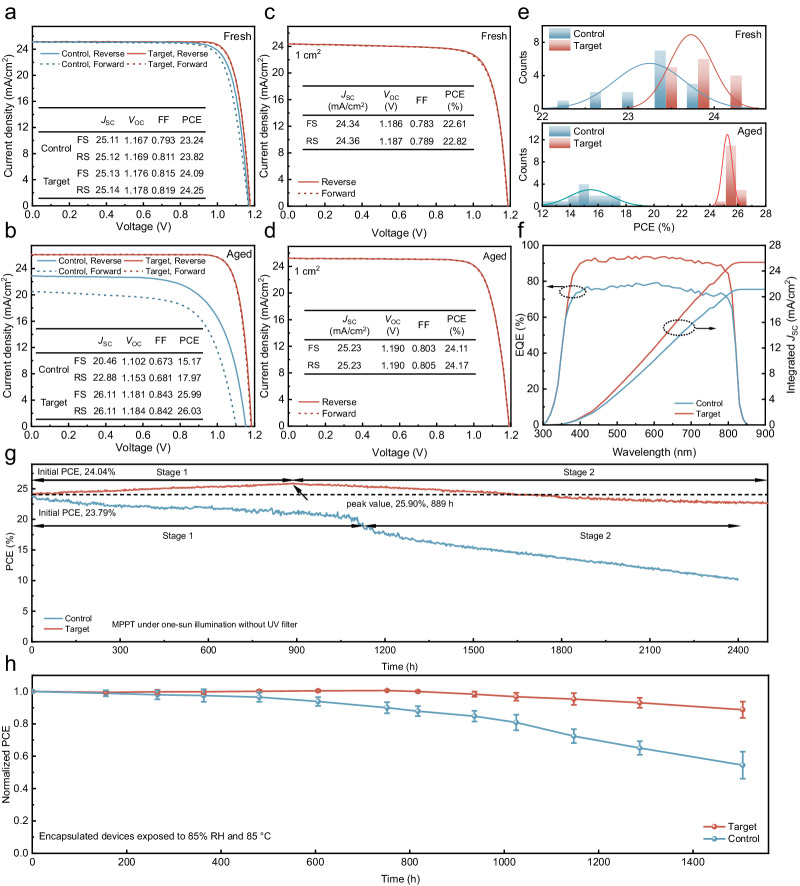
which replaces the previous incorrect version: