# Supplementary material for: Silver coordination-induced n-doping of PCBM for stable and efficient inverted perovskite solar cells
Source: Nat Commun. 2024 Jun 10;15:4922. doi: 10.1038/s41467-024-49395-7 (PMC11164978; doi:10.1038/s41467-024-49395-7)
Supplement: Supplementary file 1 — Supplementary Information [file 41467_2024_49395_MOESM1_ESM.pdf]

Supplementary Information

Silver coordination-induced n-doping of PCBM for stable and efficient inverted perovskite solar cells

Cheng Gong[1,4], Haiyun Li[1,4], Huaxin Wang[1,4], Cong Zhang[1], Qixin Zhuang[1], Awen Wang[2], Zhiyuan Xu[1], Wensi Cai[1], Ru Li[1], Xiong Li[2,*] and Zhigang Zang[1,3*]

[1]Key Laboratory of Optoelectronic Technology & Systems (Ministry of Education), Chongqing University, Chongqing 400044, China

[2]Wuhan National Laboratory for Optoelectronics, Huazhong University of Science and Technology, Wuhan 430074, Hubei, China

[3]College of Information Science and Engineering, Yanshan University, Qinhuangdao 066004, China

[4]These authors contributed equally: Cheng Gong, Haiyun Li, Huaxin Wang

*Correspondence to: xiongli@hust.edu.cn (X. L.); zangzg@cqu.edu.cn (Z. Z.)

Table of Contents

1. Supplementary Figures **3**

2. Supplementary Tables **50**

3. Supplementary References **58**

1. Supplementary Figures

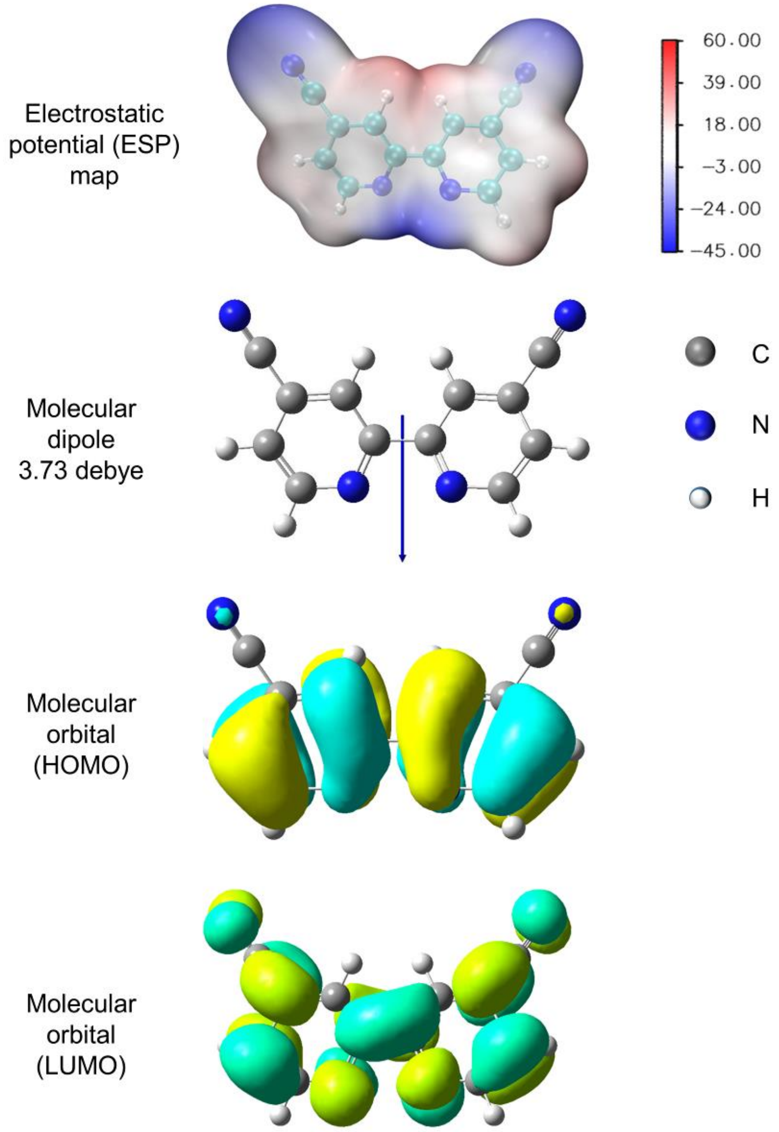

Supplementary Fig. 1. Density functional theory (DFT) calculation results for bipyridine derivative 4,4'-dicyano-2,2'-bipyridine (DCBP).

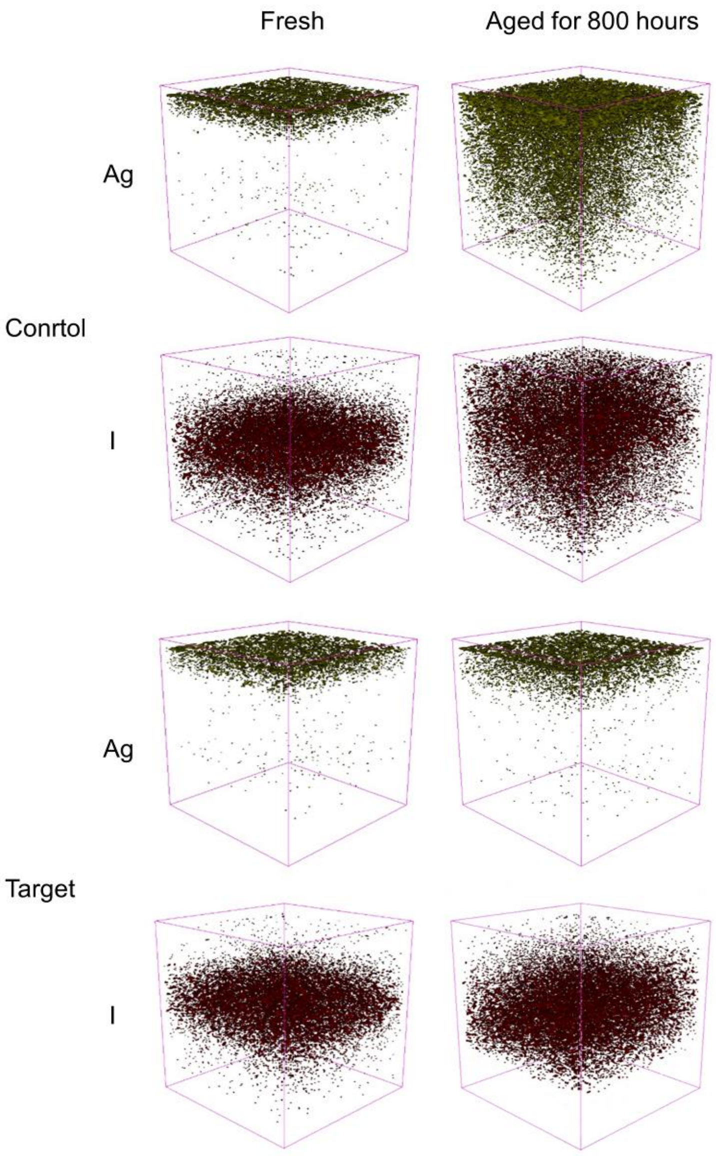

Supplementary Fig. 2. Three-dimensional distribution maps of iodine and Ag within the control and target devices following 800 h of continuous simulated AM1.5 illumination aging.

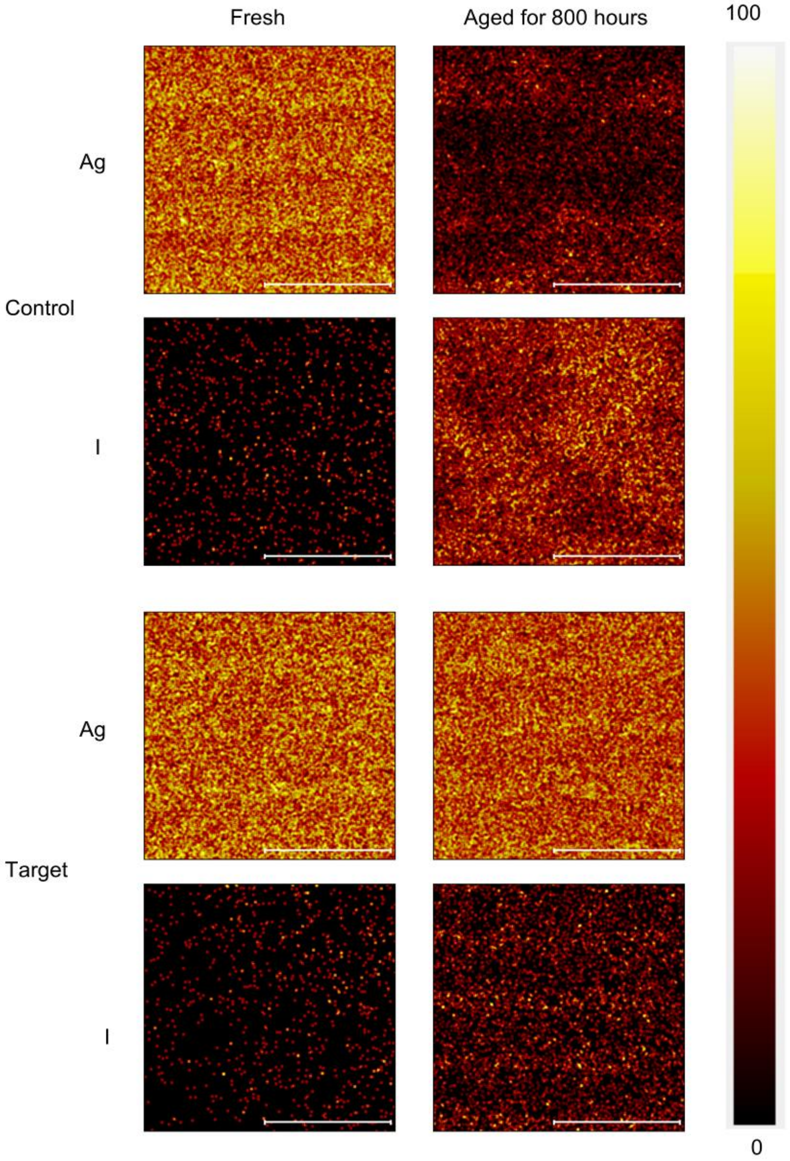

Supplementary Fig. 3. Time-of-flight secondary ion mass spectrometry (ToF-SIMS) analysis was conducted on iodine and Ag in the PCBM of perovskite solar cells (PSCs) with the structure HTL/Perovskite/PCBM/Ag for the control and HTL/Perovskite/PCBM@DCBP/Ag for the target, respectively. The devices were subjected to 800 hours of continuous simulated AM1.5 illumination. The Ag electrodes were deposited with a thickness of less than 5 nm. The scale bar is 10 μm.

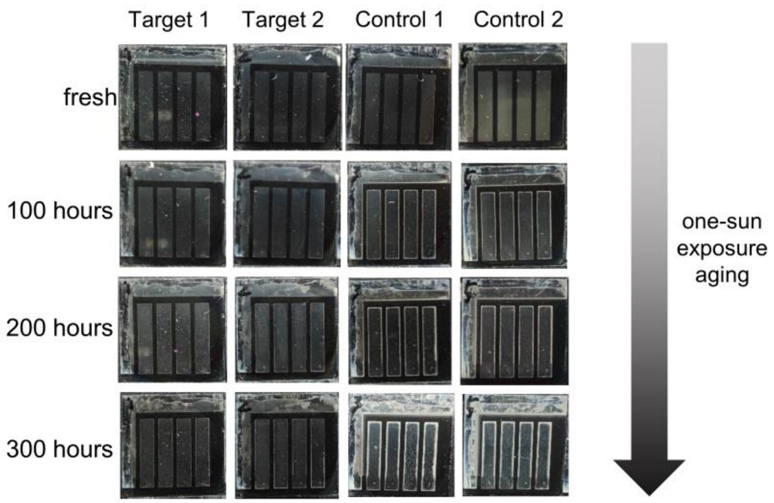

Supplementary Fig. 4. A series of photographs depicting the continuous one-sun exposure aging of both control and target devices under a nitrogen environment.

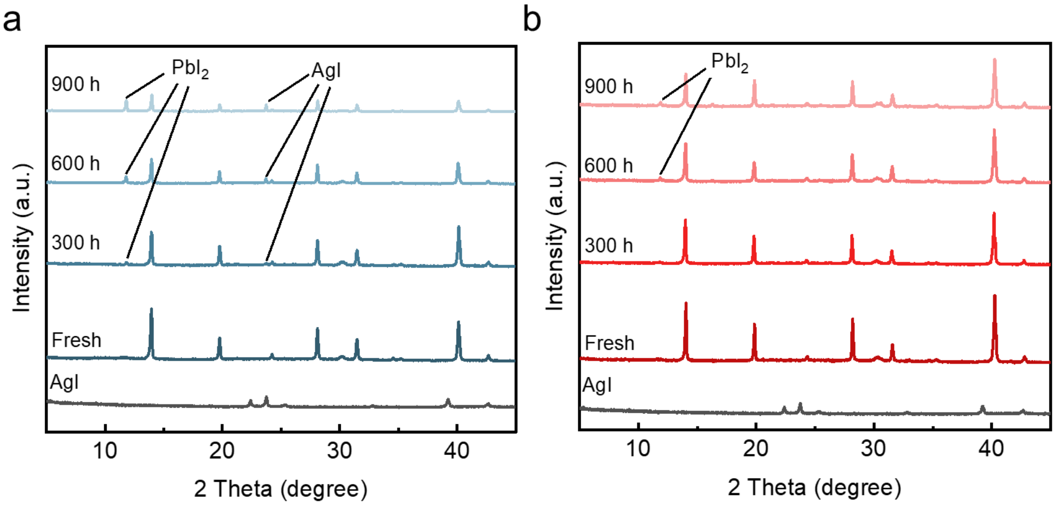

Supplementary Fig. 5. XRD patterns as a function of time for the control (**a**) and target (**b**) perovskite/PCBM/Ag films under continuous simulated AM1.5 illumination. The Ag electrodes present were removed using Kapton tape after aging.

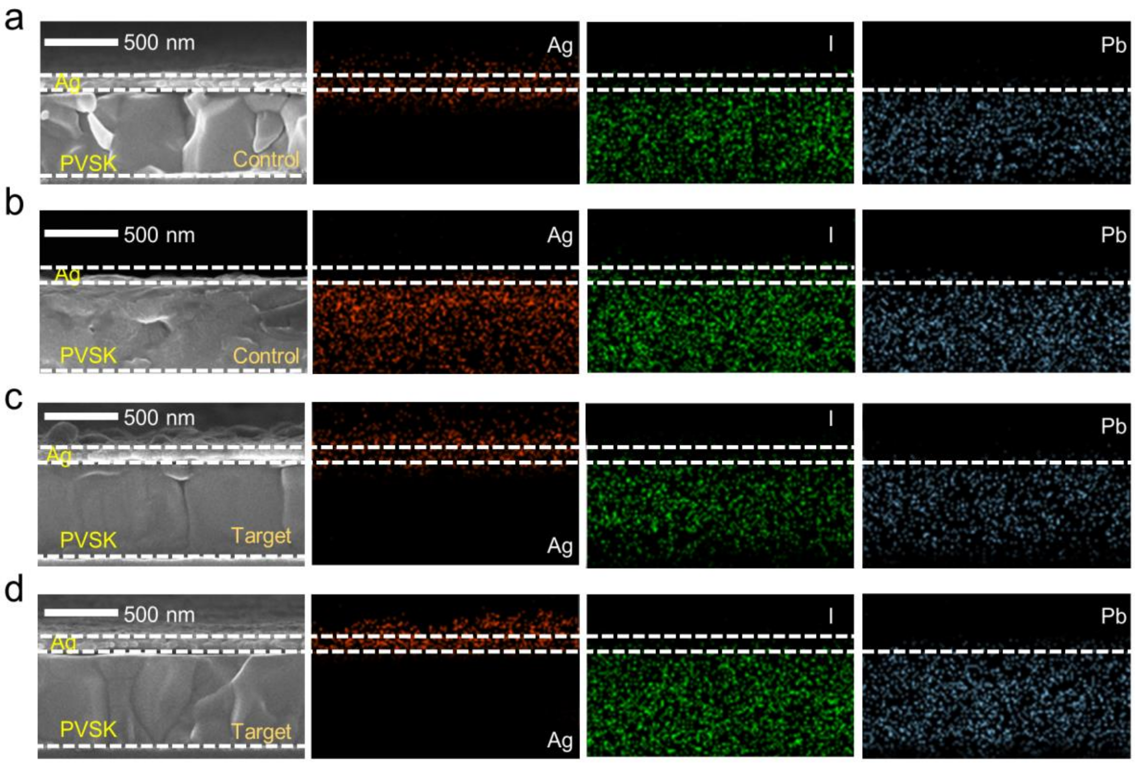

Supplementary Fig. 6. Cross-sectional morphology of the devices in their fresh state (**a**, **c**) and after 1,100 hours of continuous simulated AM1.5 illumination aging (**b**, **d**), along with EDX mapping of Ag, I, and Pb.

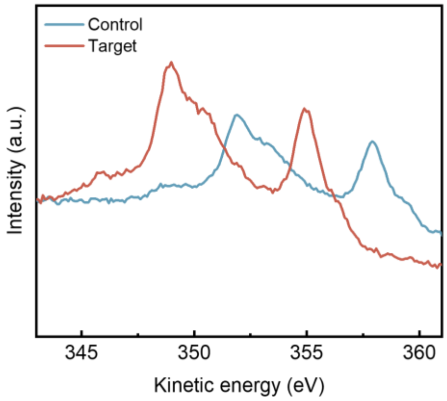

Supplementary Fig. 7. The AES spectra of Ag are obtained for pristine PCBM/Ag and PCBM@DCBP/Ag films after continuous simulated AM1.5 illumination aging for 800 hours. The Ag electrodes present were removed using Kapton tape after aging.

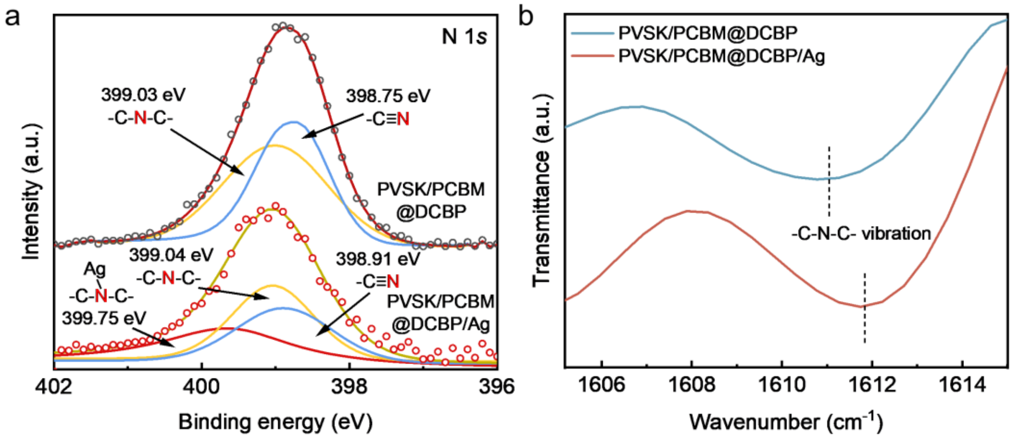

Supplementary Fig. 8. a, XPS spectra of N 1*s* for the PVSK/PCBM@DCBP and PVSK/PCBM@DCBP/Ag films after continuous simulated AM1.5 illumination aging for 800 hours. **b**, Fourier transforms infrared (FTIR) spectra of PVSK/PCBM@DCBP and PVSK/PCBM@DCBP/Ag films. The Ag electrodes present in both of these post-aging configurations were removed using Kapton tape.

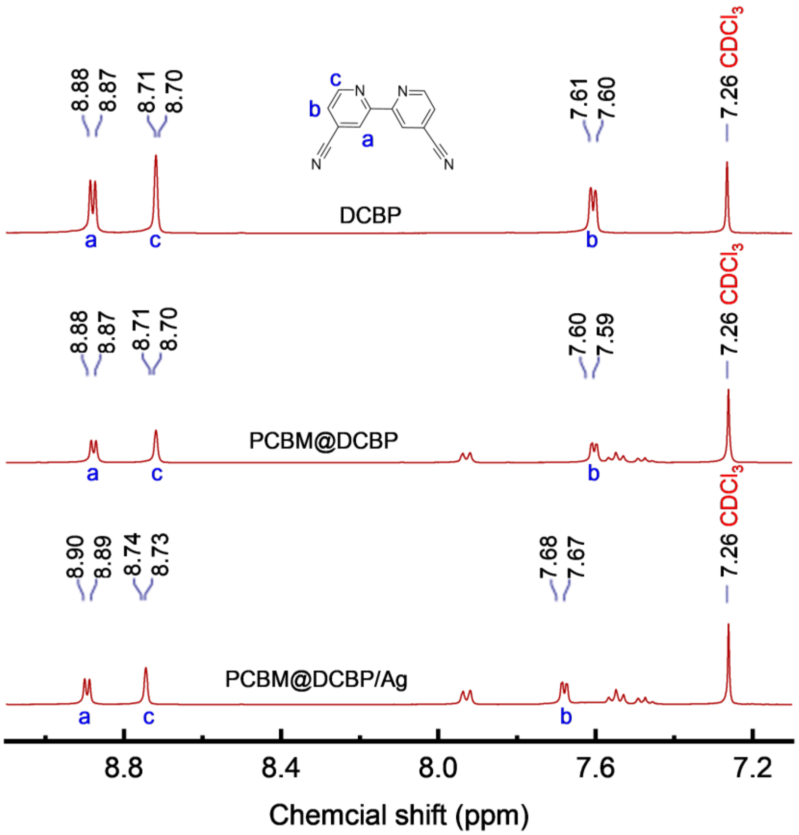

Supplementary Fig. 9. Nuclear magnetic resonance hydrogen spectra (^{1}H NMR) spectra of DCBP, PCBM@DCBP and PCBM@DCBP/Ag in $CDCl_3$.

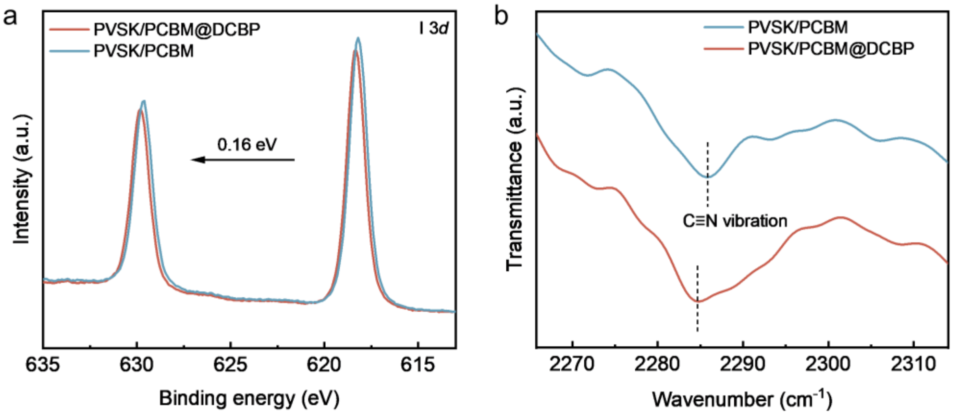

Supplementary Fig. 10. a, XPS spectra of I 3*d* for the PVSK/PCBM and PVSK/PCBM@DCBP films after continuous simulated AM1.5 illumination aging for 800 hours. **b**, FTIR spectra of PVSK/PCBM and PVSK/PCBM@DCBP films.

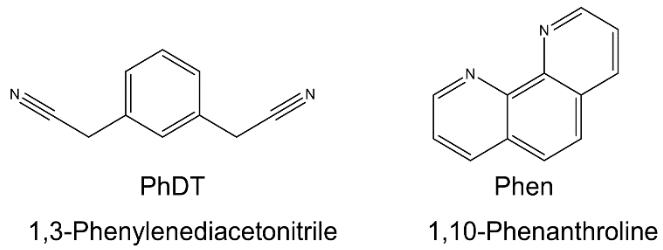

Supplementary Fig. 11. The molecular structures of PhDT and Phen.

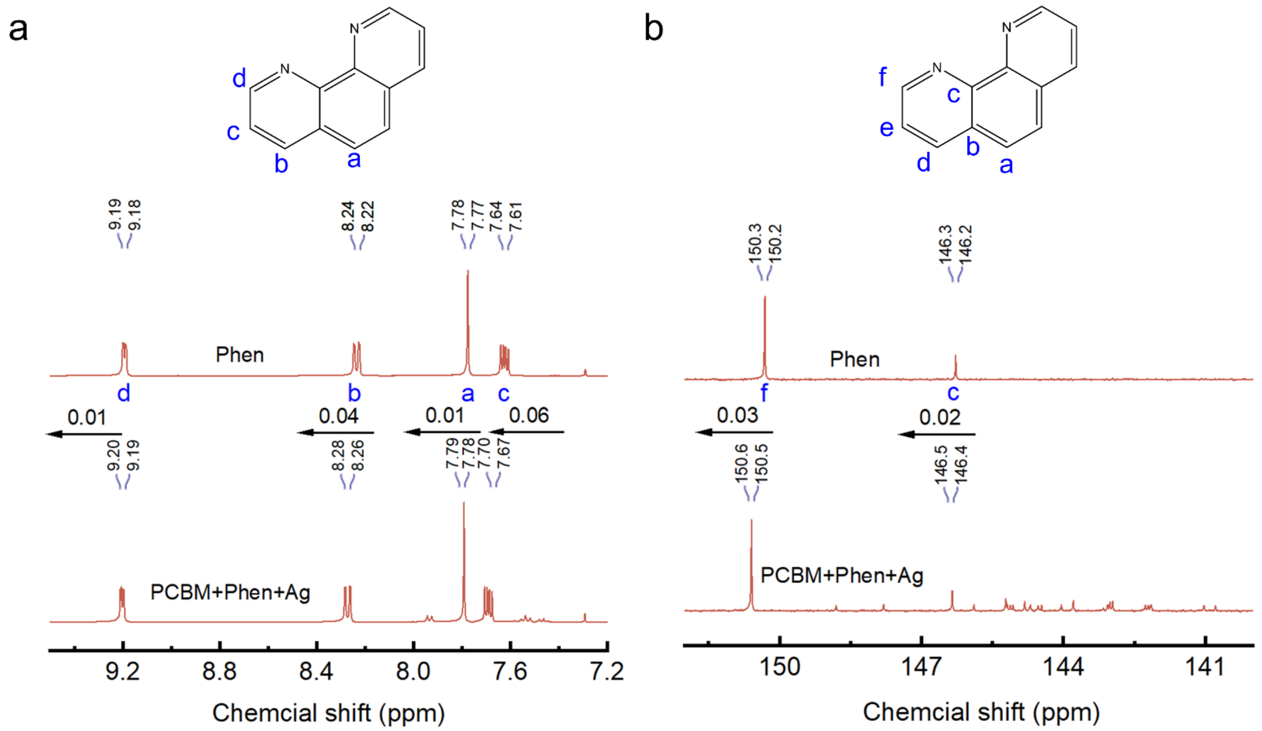

Supplementary Fig. 12. a, ^{1}H NMR spectra of the pure Phen molecule and PCBM+Phen+Ag solutions. **b**, ^{13}C NMR spectra of the pure Phen molecule and PCBM+Phen+Ag solutions. Ag powder is added to the deuterated reagent containing PCBM and Phen and stirred for six hours to allow complete reaction of Phen with Ag. The resulting solution is then filtered to obtain the PCBM+Phen+Ag solution. Compared to the Phen sample, the chemical state of the hydrogen atoms on the pyridine ring of Phen in the PCBM+Phen+Ag sample has shifted (a: 0.01 ppm; b: 0.04 ppm; c: 0.06 ppm; d: 0.01 ppm) (Supplementary Fig. 12a). Additionally, compared to the Phen sample, the chemical state of the carbon atoms on the pyridine ring of Phen in the PCBM+Phen+Ag sample has also shifted (c: 0.02 ppm; f: 0.03 ppm) (Supplementary Fig. 12b). The results of ^{1}H NMR spectra and ^{13}C NMR indicate that the pyridine functional group in Phen can effectively form coordination reaction with Ag, resulting in a reduction in the electron density around the hydrogen and carbon atoms on the pyridine ring, leading to the observed shift phenomenon.

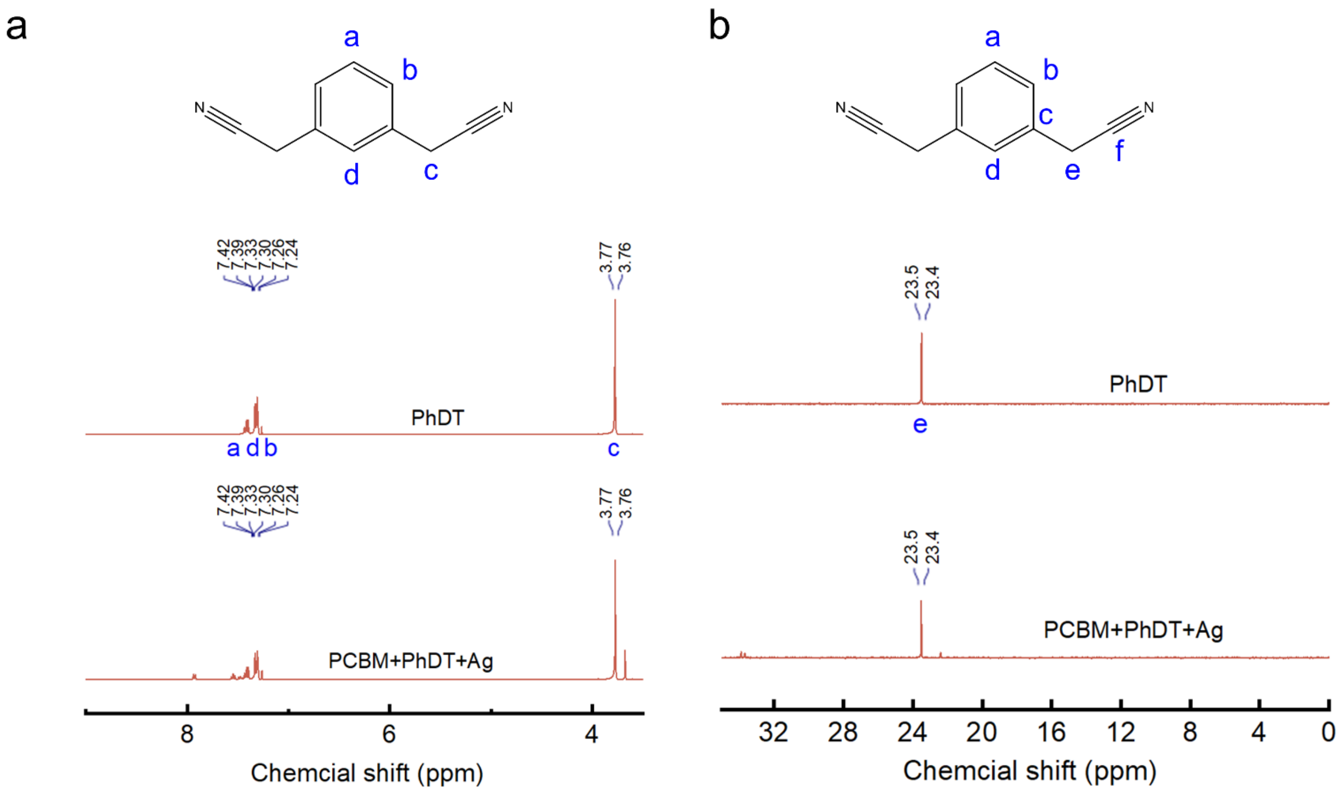

Supplementary Fig. 13. a, ^{1}H NMR spectra of the pure PhDT molecule and PCBM+PhDT+Ag solutions. **b**, ^{13}C NMR spectra of the pure PhDT molecule and PCBM+PhDT+Ag solutions. Ag powder is added to the deuterated reagent containing PCBM and PhDT and stirred for six hours to allow complete reaction of PhDT with Ag. The resulting solution is then filtered to obtain the PCBM+PhDT+Ag solution. In comparing the ^{1}H NMR and ^{13}C NMR spectra of PhDT and PCBM+PhDT+Ag samples, it is observed that the chemical states of hydrogen and carbon atoms around the cyanide group remain unchanged (Supplementary Fig. 13). This indicates that there is no coordination reaction between the cyanide group in PhDT and Ag.

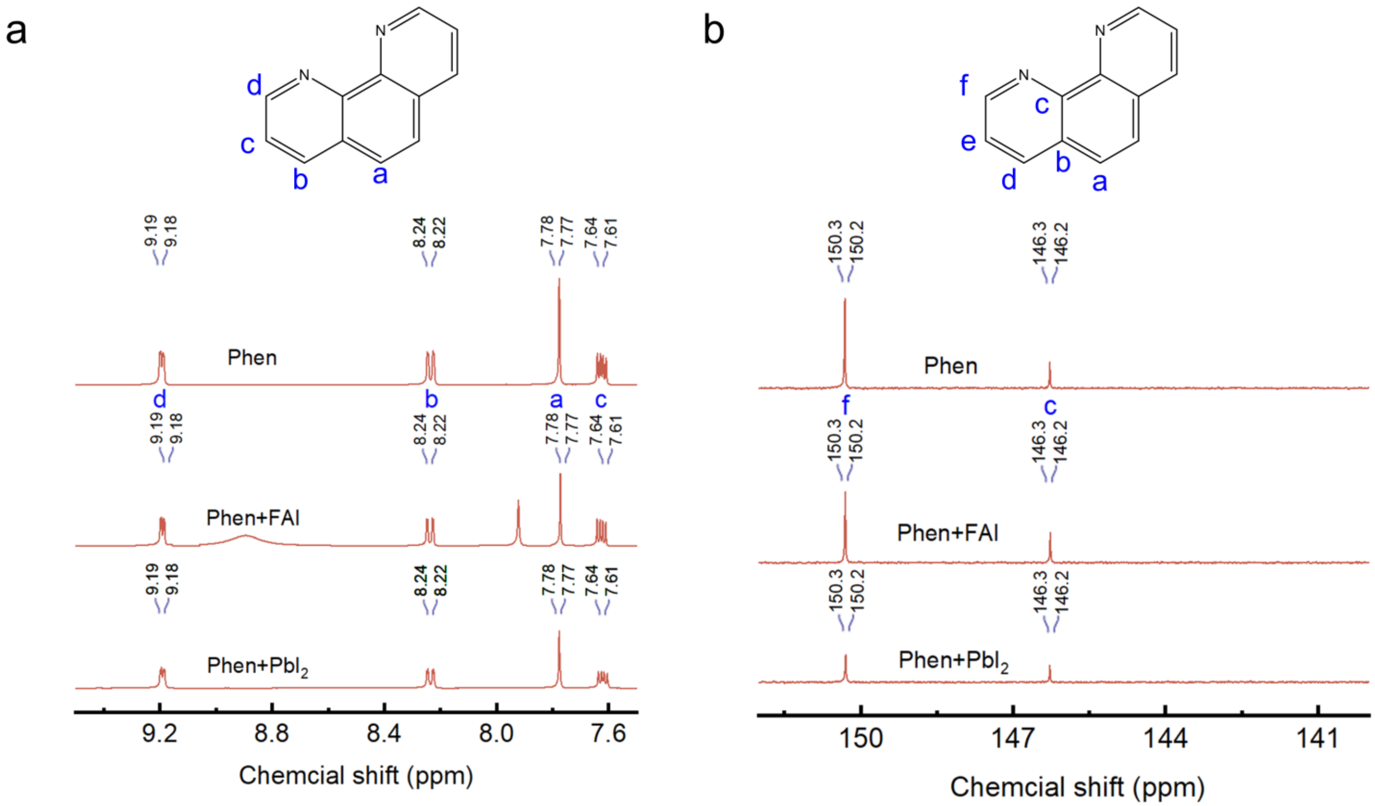

Supplementary Fig. 14. a, ^{1}H NMR spectra of the pure Phen molecule, Phen+FAI and Phen+PbI_2 solutions. **b**, ^{13}C NMR spectra of the pure Phen molecule, Phen+FAI and Phen+PbI_2 solutions. The results indicate that the chemical states of hydrogen and carbon atoms in Phen remain unchanged, suggesting no interaction between the iodine atoms in FAI/PbI_2 and Phen.

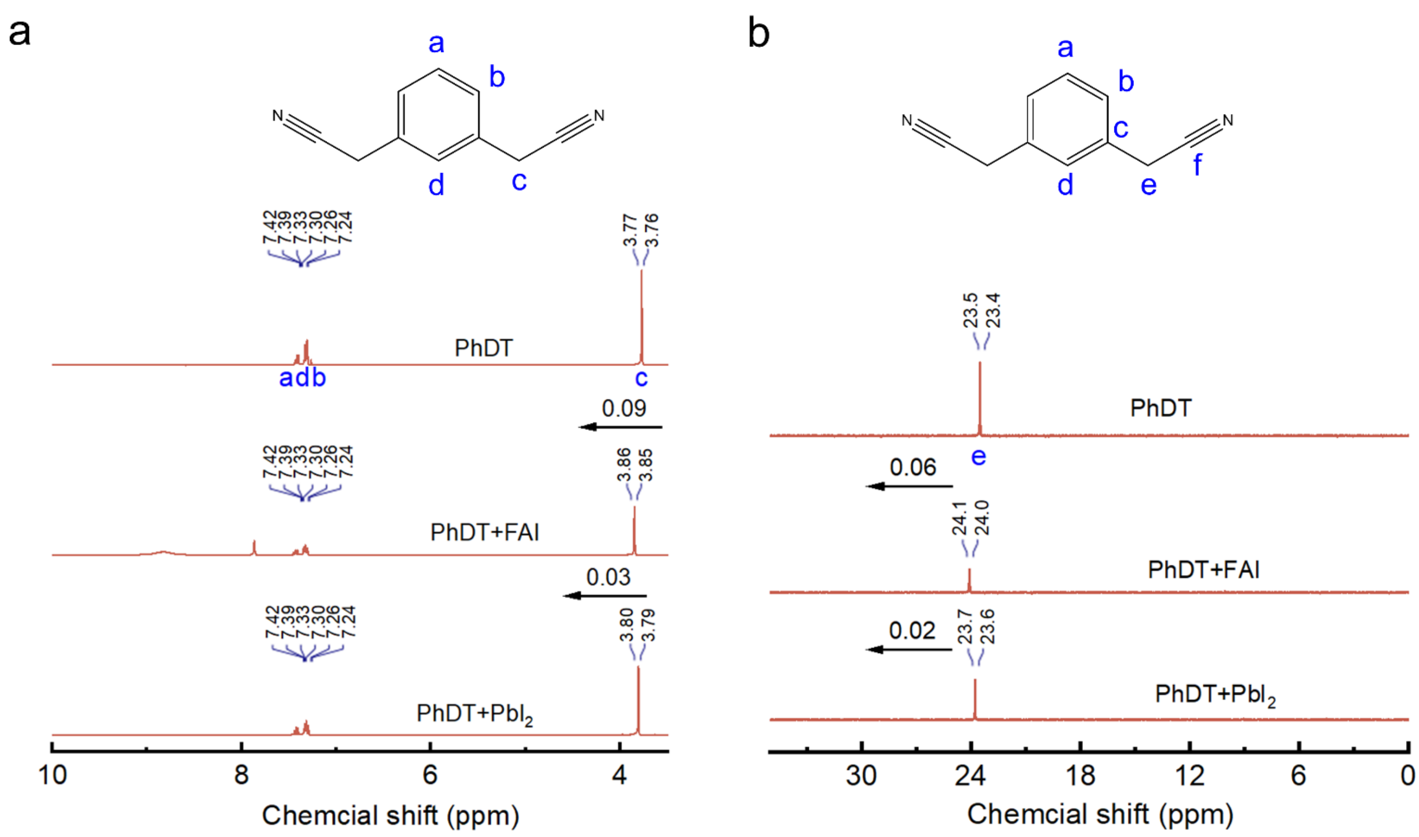

Supplementary Fig. 15. a, ^{1}H NMR spectra of the pure PhDT molecule, PhDT+FAI and PhDT+PbI_2 solutions. **b**, ^{13}C NMR spectra of the pure PhDT molecule, PhDT+FAI and PhDT+PbI_2 solutions. When PhDT is mixed separately with FAI or PbI_2, there is a significant shift in the chemical states of the hydrogen and carbon atoms around the cyanide group compared PhDT sample (Supplementary Fig. 15). This indicates that the cyano group in PhDT is capable of exerting strong interactions with FAI or PbI_2.

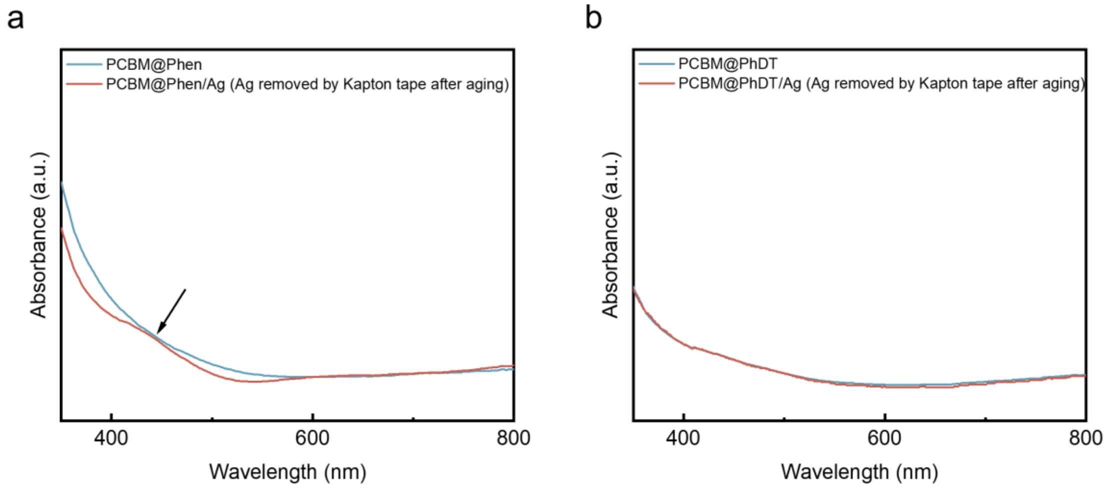

Supplementary Fig. 16. a, UV–vis absorption spectra of the PCBM@Phen film. **b**, UV–vis absorption spectra of the PCBM@PhDT film. Further, PCBM solutions containing Phen and PhDT are prepared into thin films via spin-coating method. Subsequently, approximately 100 nm of Ag layer is deposited onto the thin films through thermal evaporation. The films are then aged for 800 hours under AM1.5 illumination to ensure a sufficient amount of Ag participates in the coordination reaction. The Ag layer is peeled off by Kapton tape to facilitate the smooth progress of UV-visible spectroscopy testing on the PCBM thin film modified by Phen or PhDT. In Supplementary Fig. 16a, an additional peak appears at 430 nm in the PCBM@Phen/Ag film (Ag removed by Kapton tape after aging) compared to the PCBM@Phen film. Furthermore, the UV spectra of PCBM@Phen/Ag (Ag removed by Kapton tape after aging) are identical to those of PCBM@PhDT, with no additional peaks observed (Supplementary Fig. 16b). This suggests that the pyridine groups in Phen can coordinate with Ag, whereas the cyano group in PhDT do not exhibit coordination with Ag.

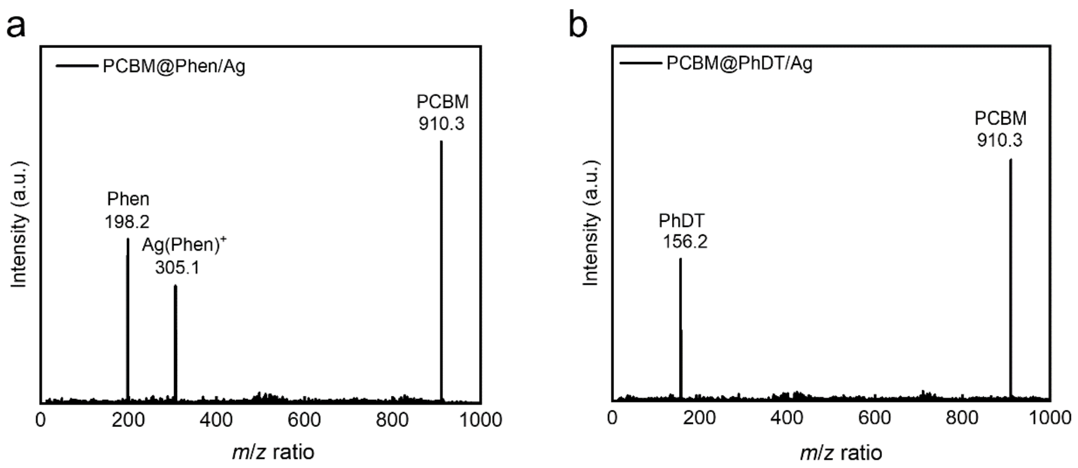

Supplementary Fig. 17. a, Time-of-flight mass spectrum of PCBM@Phen/Ag film after continuous simulated AM1.5 illumination to aging for 800 h. The Ag electrodes present were removed using Kapton tape after aging. **b**, Time-of-flight mass spectrum of PCBM@PhDT/Ag film after continuous simulated AM1.5 illumination to aging for 800 h. The Ag electrodes present were removed using Kapton tape after aging. The time-of-flight mass spectrum of the PCBM@Phen/Ag and PCBM@PhDT/Ag samples is depicted in Supplementary Fig. 17. Three distinct peaks are observed in Supplementary Fig. 17a, corresponding to the Phen monomer, PCBM, and $Ag(Phen)^+$, indicating the formation of new chelates resulting from coordination reactions between Ag and Phen within the thin film. Conversely, Supplementary Fig. 17b displays only two prominent peaks, attributed to PhDT monomer and PCBM, suggesting that the cyano group in PhDT do not engage in coordination reactions with Ag.

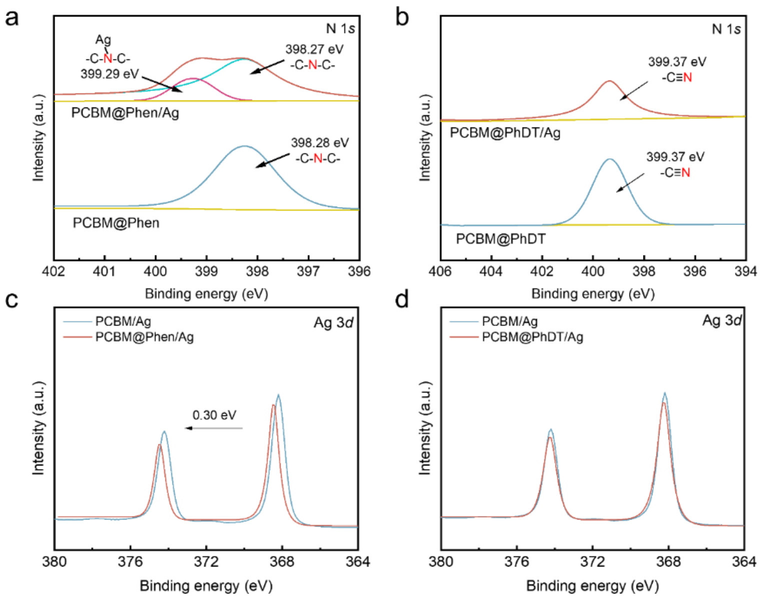

Supplementary Fig. 18. **a**, XPS spectra of N 1*s* for the PCBM@Phen/Ag and PCBM@Phen films after continuous simulated AM1.5 illumination aging for 800 hours. **b**, XPS spectra of N 1*s* for the PCBM@PhDT/Ag and PCBM@PhDT films after continuous simulated AM1.5 illumination aging for 800 hours. **c**, XPS spectra of Ag 3*d* for the PCBM/Ag and PCBM@Phen/Ag films after continuous simulated AM1.5 illumination aging for 800 hours. **d**, XPS spectra of Ag 3*d* for the PCBM/Ag and PCBM@PhDT/Ag films after continuous simulated AM1.5 illumination aging for 800 hours. The Ag electrodes present in both of these post-aging configurations were removed using Kapton tape. The N 1*s* XPS spectra of the PCBM@Phen/Ag sample can be divided into two peaks, corresponding to the N atoms in the pyridine functional group and the N atoms in the complex formed between the pyridine functional group and Ag (Supplementary Fig. 18a). Combined with Supplementary Fig. 18c, it can be observed that the XPS spectra of Ag 3*d* in the PCBM@Phen/Ag sample exhibit significant shifts compared with PCBM/Ag sample. This indicates that the pyridine functional group is capable of forming effective coordination reactions with Ag. However, the N 1*s* XPS spectra of PCBM@PhDT/Ag sample show only one peak, and its binding energy position remains unchanged compared to the PCBM@PhDT sample (Supplementary Fig. 18b). Similarly, the Ag 3*d* XPS spectra of PCBM@PhDT/Ag sample also show no significant changes compared to the PCBM@PhDT sample (Supplementary Fig. 18d). This indicates that the cyano groups in PhDT do not interact with Ag.

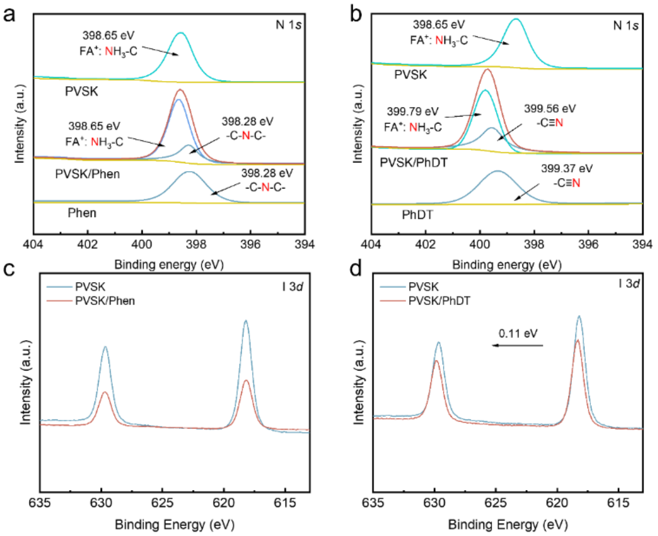

Supplementary Fig. 19. a, XPS spectra of N 1*s* for the PVSK, PVSK/Phen and Phen films after continuous simulated AM1.5 illumination aging for 800 hours. **b**, XPS spectra of N 1*s* for the PVSK, PVSK/PhDT and PhDT films after continuous simulated AM1.5 illumination aging for 800 hours. **c**, XPS spectra of I 3*d* for the PVSK and PVSK/Phen films after continuous simulated AM1.5 illumination aging for 800 hours. **d**, XPS spectra of I 3*d* for the PVSK and PVSK/PhDT films after continuous simulated AM1.5 illumination aging for 800 hours. The Ag electrodes present in both of these post-aging configurations were removed using Kapton tape. In PVSK/Phen samples, the N 1*s* XPS spectrum exhibits two peaks, positioned at 398.65 eV and 398.28 eV respectively, corresponding to the N atoms in FA^+ and the pyridine moiety of Phen (Supplementary Fig. 19a). The binding energy of N atoms in FA^+ in PVSK is located at 398.65 eV, while in Phen, the binding energy of N atoms in the pyridine functional group is at 398.28 eV. Conversely, the I 3*d* XPS spectrum in PVSK/Phen samples shows no significant changes compared to PVSK samples (Supplementary Fig. 19c). This suggests that Phen does not form strong interactions with the components in PVSK. In PVSK/PhDT samples, the N 1s XPS spectrum exhibits two peaks at 399.79 eV and 399.56 eV, corresponding to the N atoms in FA^+ and PhDT, respectively (Supplementary Fig. 19b). The binding energy position of N atoms in FA^+ in PVSK is 398.65 eV, whereas for the N atoms in the pyridine moiety of PhDT, it is 399.37 eV. On the other hand, significant changes are observed in the I 3*d* XPS spectrum of PVSK/PhDT samples compared to PVSK alone (Supplementary Fig. 19d). This indicates that the cyanide group in PhDT can form strong interactions with iodine and FA^+.

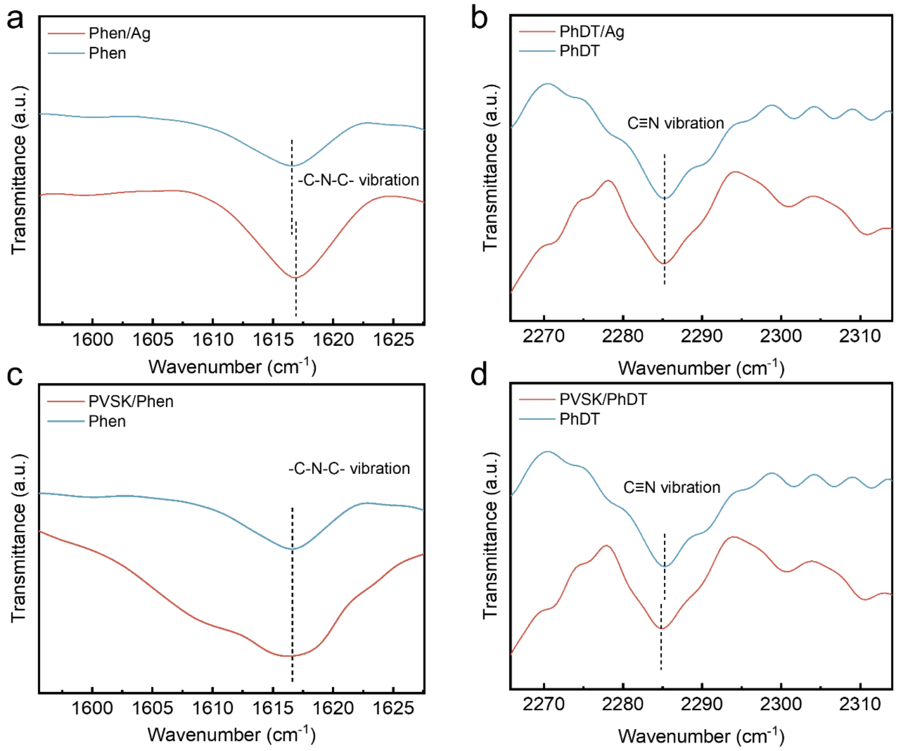

Supplementary Fig. 20. a, Fourier transforms infrared (FTIR) spectra of Phen and Phen/Ag films. **b**, FTIR spectra of PhDT and PhDT/Ag films. **c**, FTIR spectra of Phen and PVSK/Ag films. **d**, FTIR spectra of PhDT and PhDT/Ag films. **c**, FTIR spectra of PhDT and PVSK/Ag films. The Ag electrodes present in above post-aging configurations were removed using Kapton tape. The FTIR characterization further validates the aforementioned conclusions (Supplementary Fig. 20). A noticeable shift of the pyridine functional group is observed in Phen/Ag samples compared to Phen samples; however, no significant variation is observed in the pyridine functional group in PVSK/Phen compared to Phen samples. Similarly, the cyano group shows no significant change in PhDT/Ag samples compared to PhDT samples, whereas a noticeable variation in the cyano group is observed in PVSK/PhDT compared to PhDT samples. The FTIR characterization results further confirm that the pyridine functional group can coordinate with Ag without interacting with I. Conversely, the cyano group cannot coordinate with Ag but interacts strongly with I or FA.

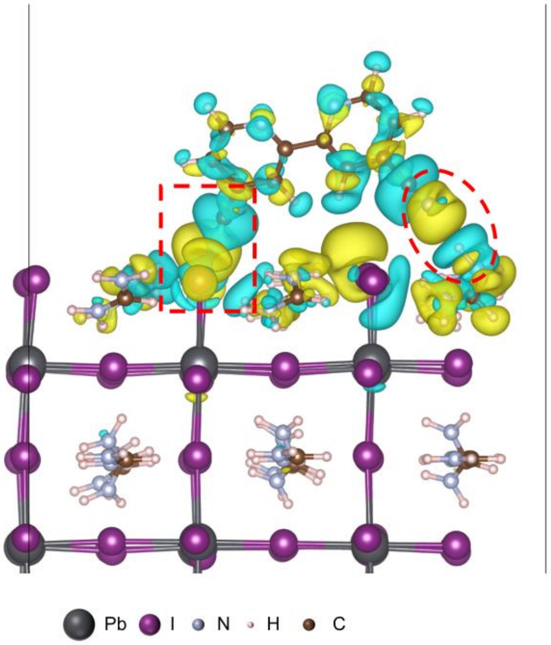

Supplementary Fig. 21. Charge density different of DCBP passivated (001) FAI-terminated $FAPbI_3$ surface with V_{FA} and V_I.

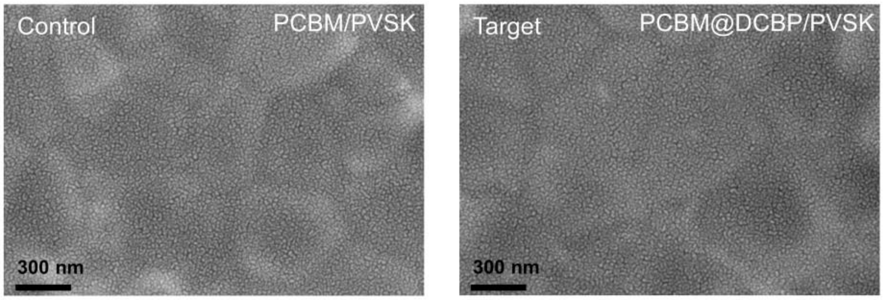

Supplementary Fig. 22. Scanning electron microscopy (SEM) images of PCBM/PVSK and PCBM@DCBP/PVSK films.

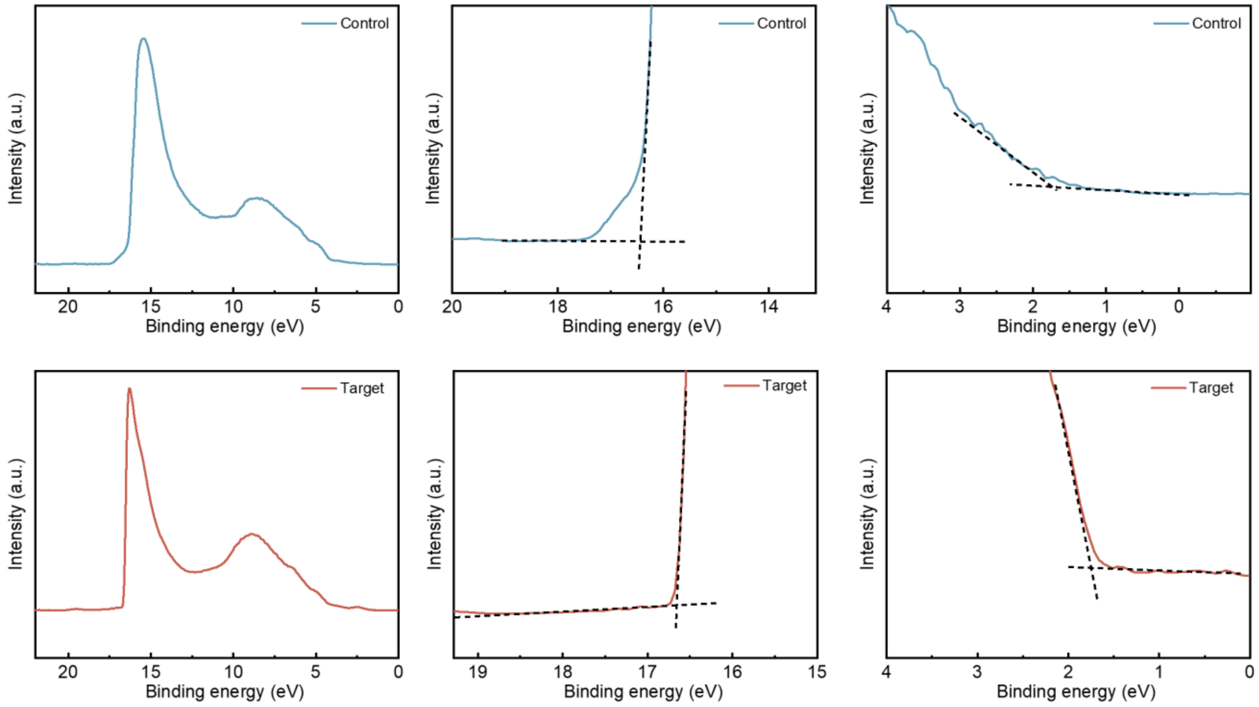

Supplementary Fig. 23. Ultraviolet photoelectron spectroscopy (UPS) measurements of the PCBM/Ag and PCBM@DCBP/Ag after continuous simulated AM1.5 illumination aging for 800 hours. The Ag electrodes present were removed using Kapton tape after aging.

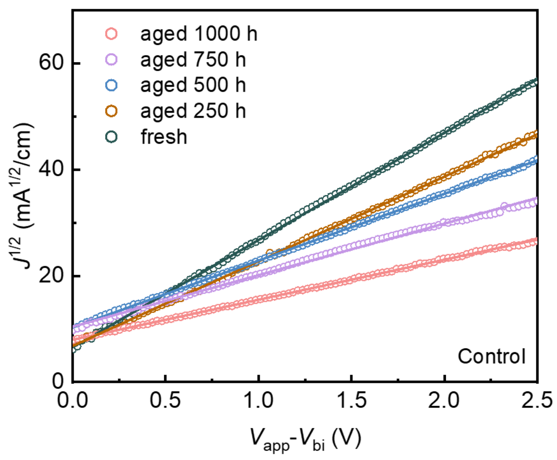

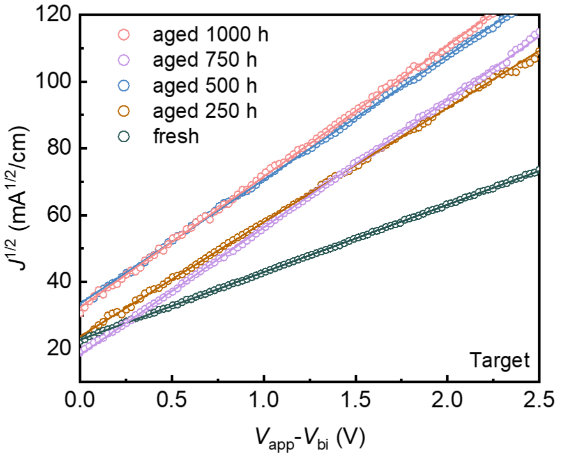

Supplementary Fig. 24. Electron mobility measurement as a function of time for PCBM/Ag and PCBM@DCBP/Ag under continuous simulated AM1.5 illumination by using the space-charge-limited current (SCLC) model with a device structure of ITO/SnO_2/PCBM without or with DCBP/Ag/SnO_2/Ag.

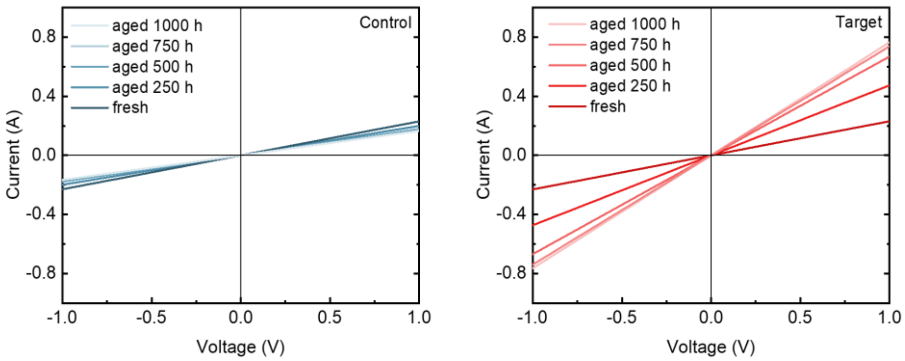

Supplementary Fig. 25. *I*–*V* curves as a function of time for PCBM and PCBM@DCBP under continuous simulated AM1.5 illumination with a device structure of ITO/PCBM without or with DCBP/Ag.

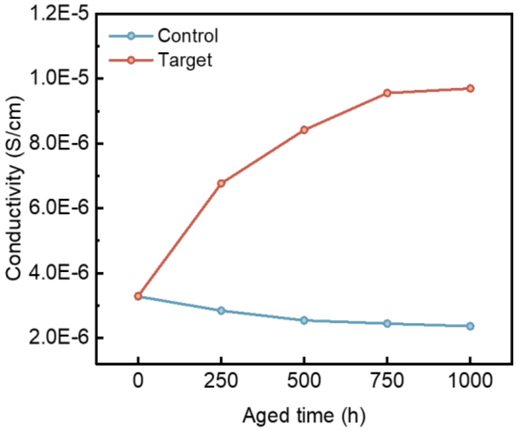

Supplementary Fig. 26. Electric conductivity as a function of time for PCBM and PCBM@DCBP under continuous simulated AM1.5 illumination.

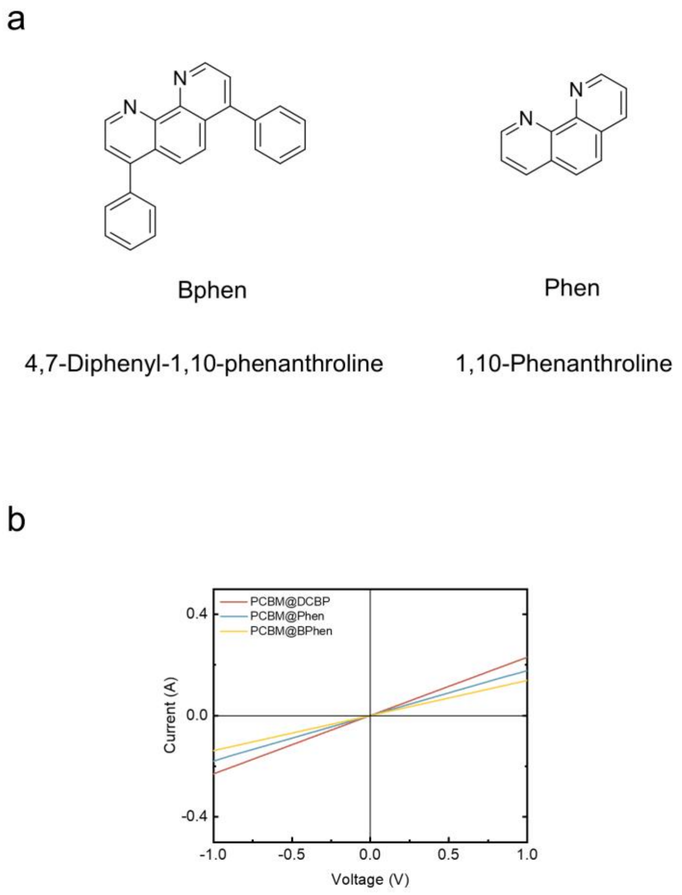

Supplementary Fig. 27. a, The molecular structures of Bphen and Phen. **b**, *I*–*V* curves of PCBM@DCBP, PCBM@Phen and PCBM@BPhen.

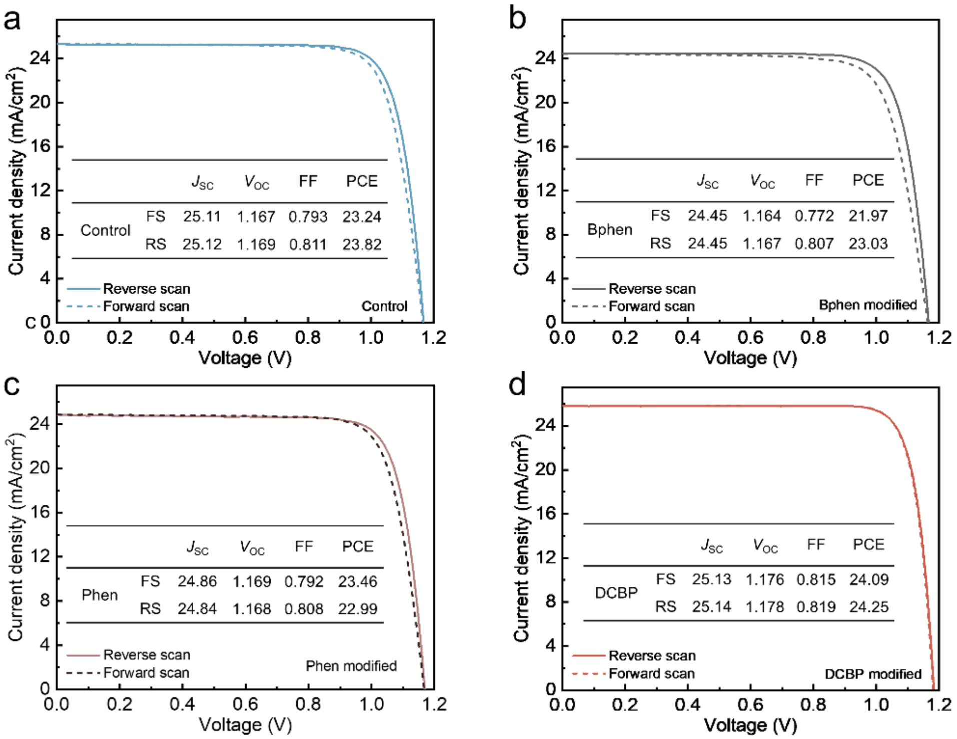

Supplementary Fig. 28. a-d *J–V* curves acquired during forward and reverse scans for both the champion control (**a**), BPhen (**b**), Phen (**c**) and DCBP (**d**) modified PSCs.

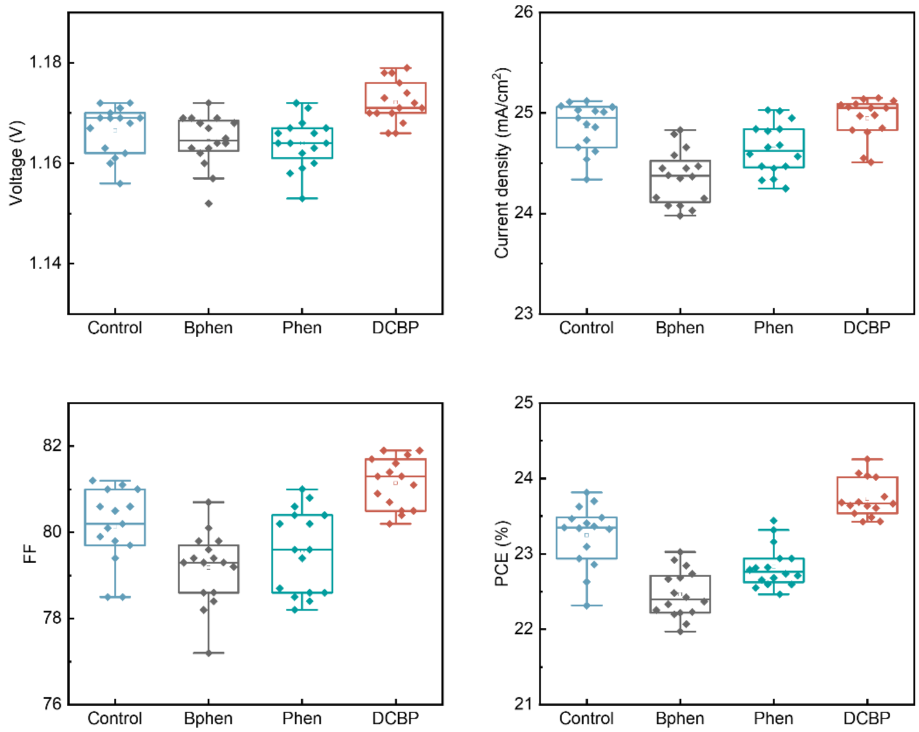

Supplementary Fig. 29. Statistical distribution diagram of the photovoltaic parameters of the control and Bphen-, Phen- and DCBP-modified PSCs at their initial states. The statistical data were obtained from 15 individual cells for each kind of device.

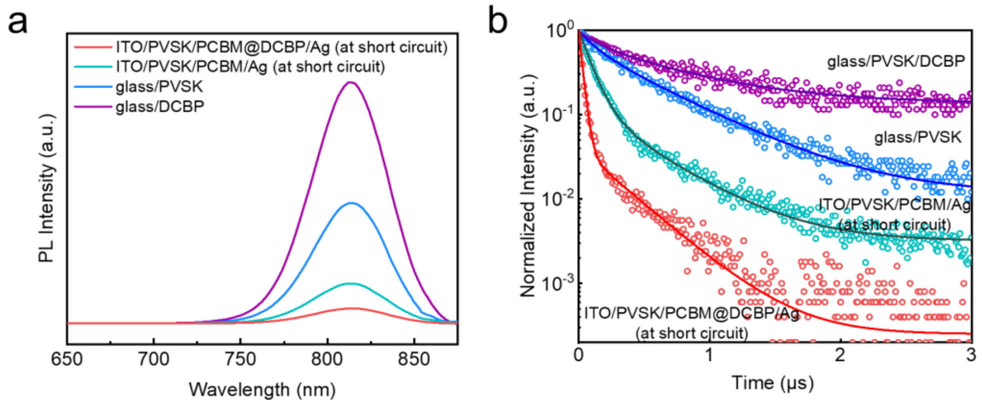

Supplementary Fig. 30. a, PL and **b**, TRPL spectra of the corresponding perovskite films with or without PCBM as an electron quencher prepared on non-conductive glass after 800 hours of continuous simulated AM1.5 illumination aging. Note that the TRPL and PL for samples with ETL were measured at a short circuit. Short-circuit conditions were established by directly connecting the Ag electrode and the ITO electrode.

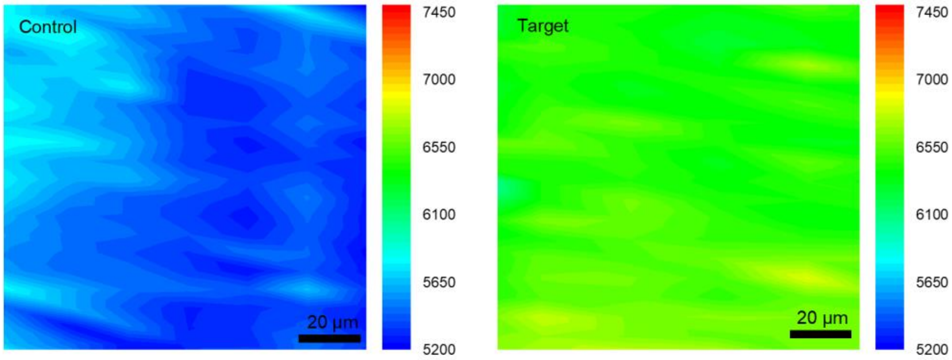

Supplementary Fig. 31. PL mapping images of the PVSK and PVSK/DCBP films deposited on non-conductive glass.

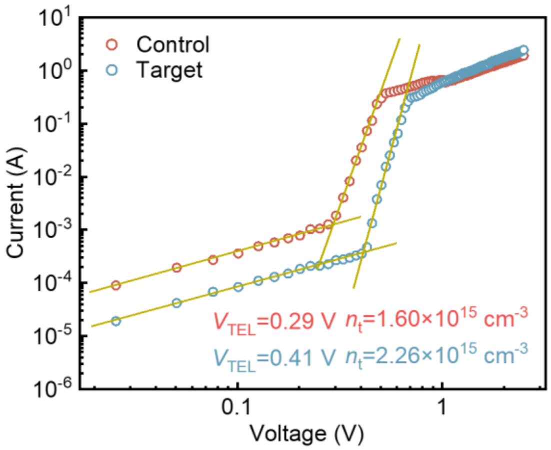

Supplementary Fig. 32. SCLC plots were obtained for the electron-only device with the structure ITO/SnO_2/PVSK/PCBM/BCP/Ag, using PCBM films both without and with DCBP.

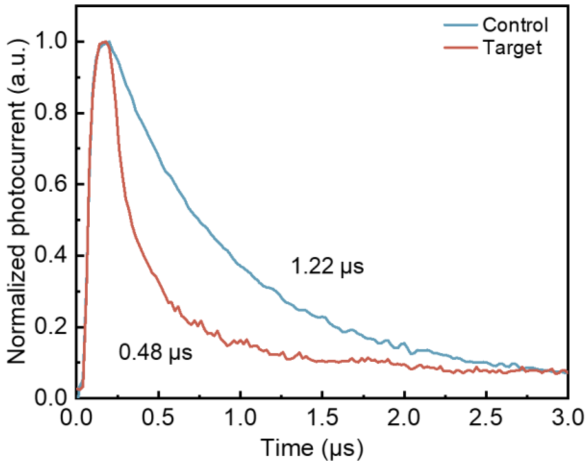

Supplementary Fig. 33. TPC for the control and modified PSCs after 800 hours of continuous simulated AM1.5 illumination aging.

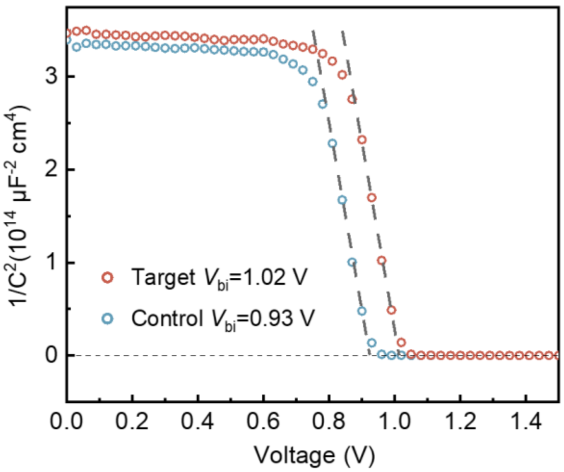

Supplementary Fig. 34. Mott–Schottky analysis for the PSCs without and with DCBP after 800 hours of continuous simulated AM1.5 illumination aging.

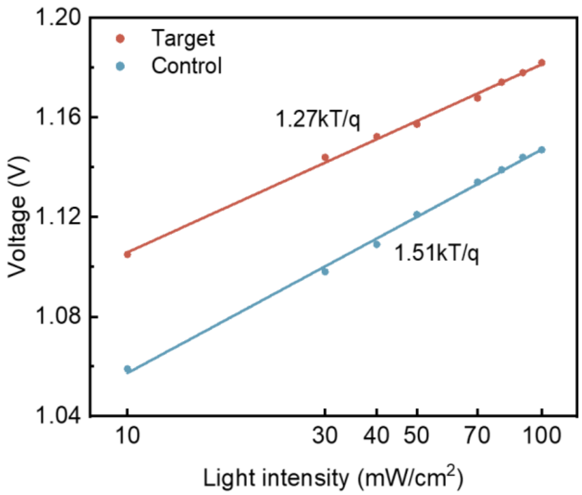

Supplementary Fig. 35. V_{OC} versus light intensity for the control and target after 800 hours of continuous simulated AM1.5 illumination aging. q: electron charge, k_B: Boltzmann constant, T: kelvin temperature.

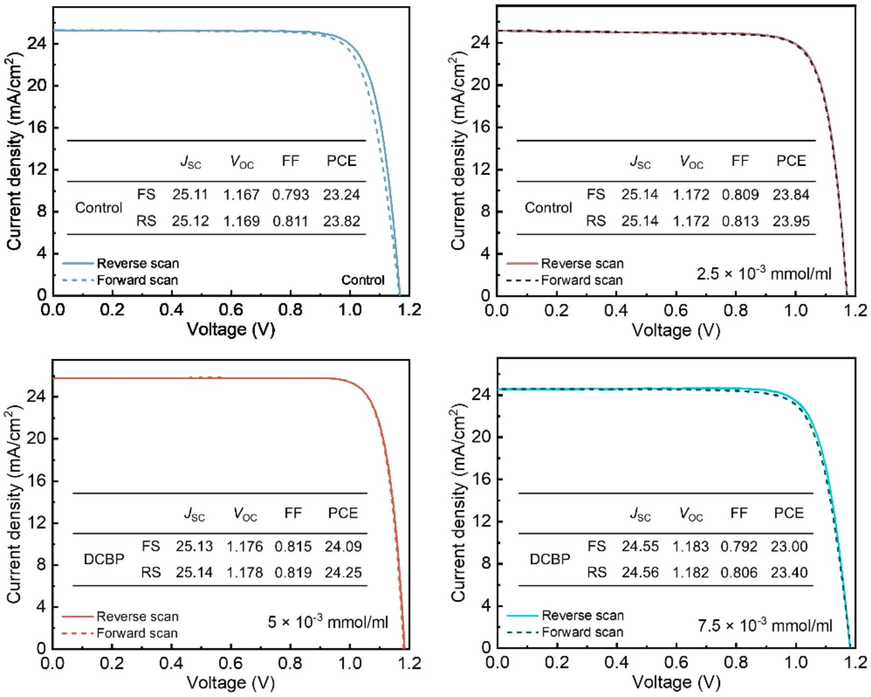

Supplementary Fig. 36. *J*–*V* curves acquired during forward and reverse scans for both the champion control and target PSCs modified by different concentration DCBP.

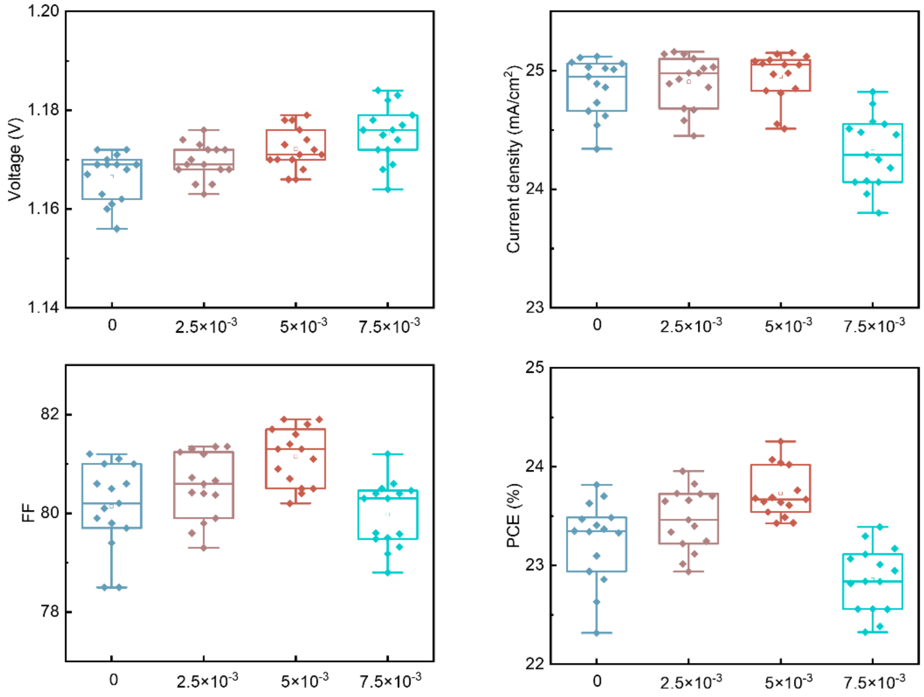

Supplementary Fig. 37. Statistical distribution diagram of the photovoltaic parameters for the PSCs modified by different molar concentration (mmol/mL) of DCBP at their initial states. The statistical data were obtained from 15 individual cells for each kind of device.

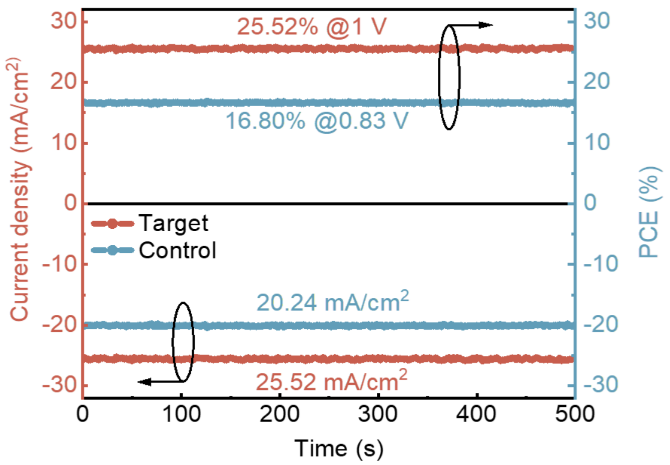

Supplementary Fig. 38. Steady-state current density and PCE versus time for the best-performing devices measured at the maximum power point after 800 hours of continuous simulated AM1.5 illumination aging.

Test and Calibration Center of New Energy Device and Module,
Shanghai Institute of Microsystem and Information Technology,
Chinese Academy of Sciences (SIMIT)

Measurement Report

Report No. 23TR102502

**Client Name**	Chongqing University
**Client Address**	No. 174 Shazheng Street, Shapingba District, Chongqing
**Sample**	Perovskite Solar Cell
**Manufacturer**	College of Optoelectronic Engineering
**Measurement Date**	25th October, 2023

**Performed by:**	Qiang Shi *Qiang shi*	**Date:** 25/10/2023
**Reviewed by:**	Wenjie Zhao *Wenjie Zhao*	**Date:** 25/10/2023
**Approved by:**	Yucheng Liu *Yucheng Liu*	**Date:** 25/10/2023

Address: No.235 Chengbei Road, Jiading, Shanghai **Post Code:**201800

E-mail: solarcell@mail.sim.ac.cn **Tel:** +86-021-69976921

The measurement report without signature and seal are not valid.
This report shall not be reproduced, except in full, without the approval of SIMIT.

Report No. 23TR102502

Sample Information

Sample Type	Perovskite solar cell
Serial No.	14-1#
Lab Internal No.	23102501-2#
Measurement Item	I-V characteristic
Measurement Environment	23.9±2.0°C,41.3±5.0%R.H

Measurement of I-V characteristic

**Reference cell**	PVM 1121
**Reference cell Type**	mono-Si, WPVS, calibrated by NREL (Certificate No. ISO 2075)
**Calibration Value/Date of Calibration for Reference cell**	144.53mA/ Feb. 2023
**Measurement Conditions**	Standard Test Condition (STC):<br>Spectral Distribution: AM1.5 according to IEC 60904-3 Ed.3,<br>Irradiance: 1000±50W/m^2, Temperature: 25±2°C
**Measurement Equipment/ Date of Calibration**	AAA Steady State Solar Simulator (YSS-T155-2M) / July.2023<br>IV test system (ADCMT 6246) / June. 2023<br>Measuring Microscope (MF-B2017C) / July.2023<br>SR Measurement system (CEP-25ML-CAS) / April.2023
**Measurement Method**	I-V Measurement:<br>Logarithmic sweep in both directions (Voc to Isc and Isc to Voc) during one flash based on IEC 60904-1:2020.<br>Spectral Mismatch factor was calculated according to IEC 60904-7 and I-V correction according to IEC 60891.
**Measurement Uncertainty**	Area: 1.0%(k=2); Isc: 1.9%(k=2); Voc: 1.0%(k=2);<br>Pmax: 2.4%(k=2); Eff: 2.5%(k=2)

上海微系统…检测专…

Report No. 23TR102502

====Measurement Results ====

	Forward Scan (Isc to Voc)	Reverse Scan (Voc to Isc)
Area	10.01 mm^2	
Isc	2.624 mA	2.624 mA
Voc	1.170 V	1.170 V
Pmax	2.554 mW	2.535 mW
Ipm	2.514 mA	2.522 mA
Vpm	1.016 V	1.005 V
FF	83.14 %	82.54 %
Eff	25.51 %	25.33 %

- Spectral Mismatch Factor: SMM=0.9950.
- Designated illumination area defined by a thin mask was measured by measuring microscope.
- Test results listed in this measurement report refer exclusively to the mentioned measured sample.
- The results apply only at the time of the test, and do not imply future performance.

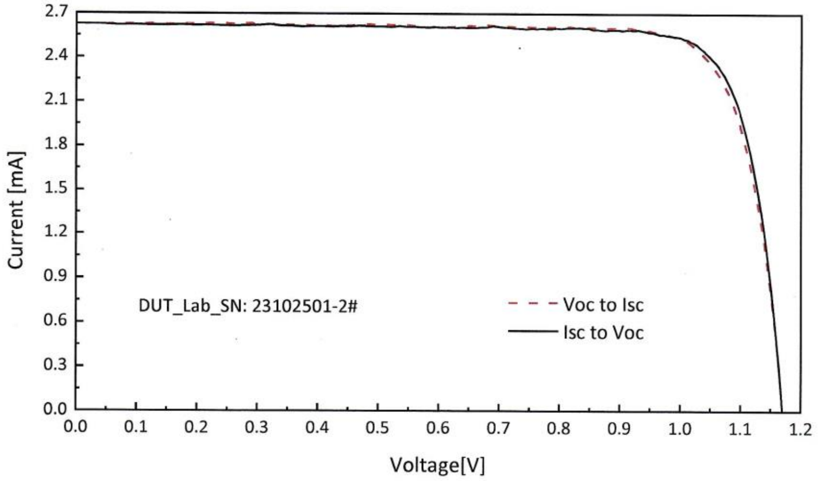

Fig.1 I-V curves of the measured sample

-------**End of Report**-------

3 / 3

Supplementary Fig. 39. Independent certification of one of the best-performing target devices by Shanghai Institute of Microsystem and Information Technology (SIMIT), Chinese Academy of Sciences. All elements in Supplementary Fig. 39 have received written approval from the copyright holders.

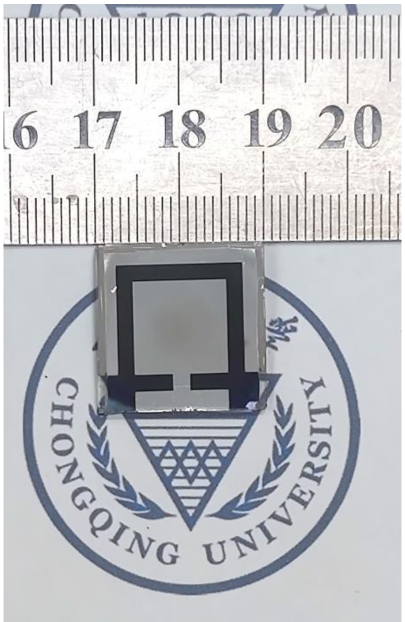

Supplementary Fig. 40. Photograph of a 1 cm² device.

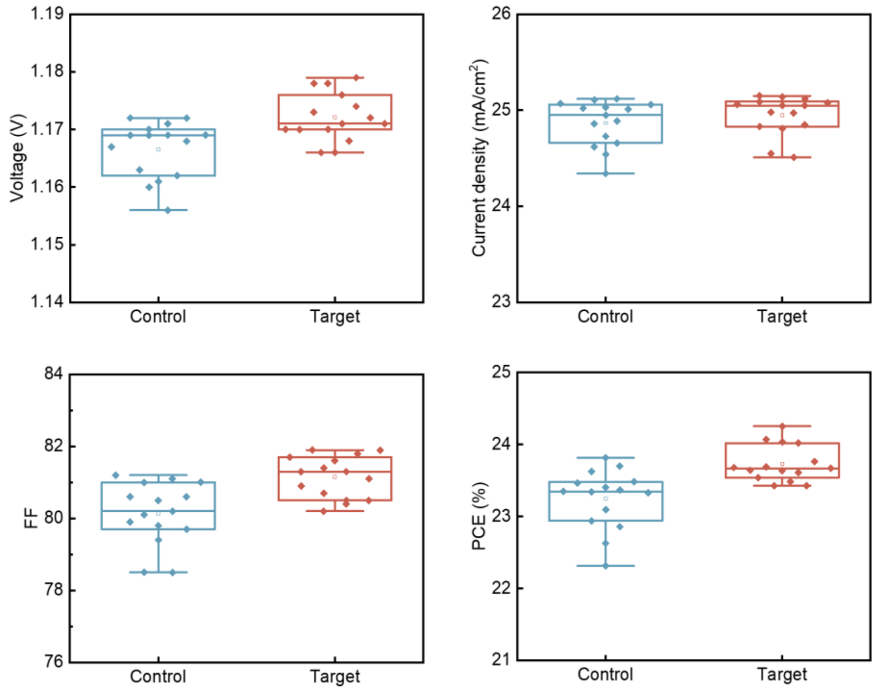

Supplementary Fig. 41. Statistical distribution diagram of the photovoltaic parameters of the fresh PSCs prepared based on PCBM or PCBM@DCBP. The statistical data were obtained from 15 individual cells for each kind of device.

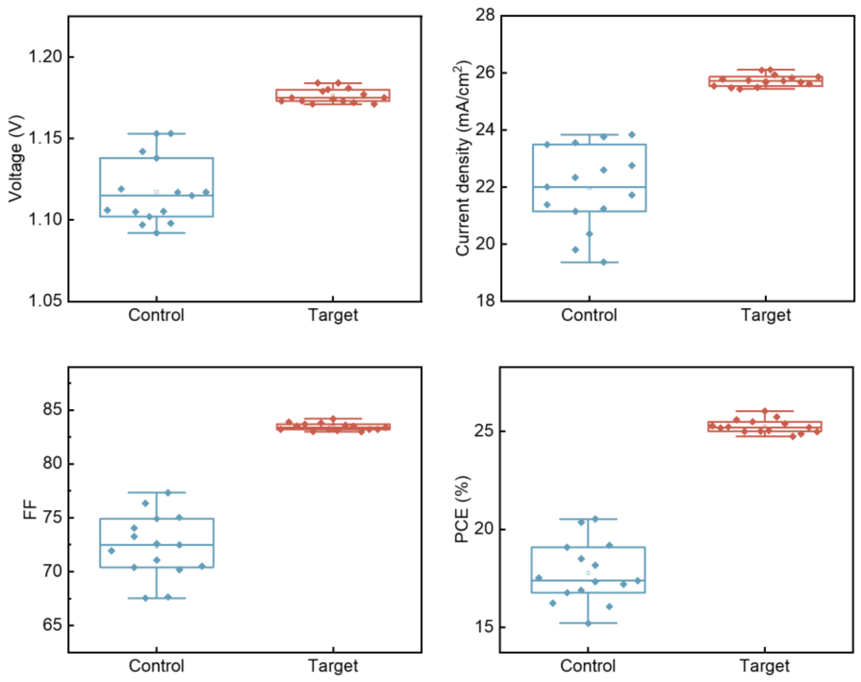

Supplementary Fig. 42. Statistical distribution diagram of the photovoltaic parameters of the PSCs prepared based on PCBM or PCBM@DCBP after 800 hours of continuous simulated AM1.5 illumination aging. The statistical data were obtained from 15 individual cells for each kind of device.

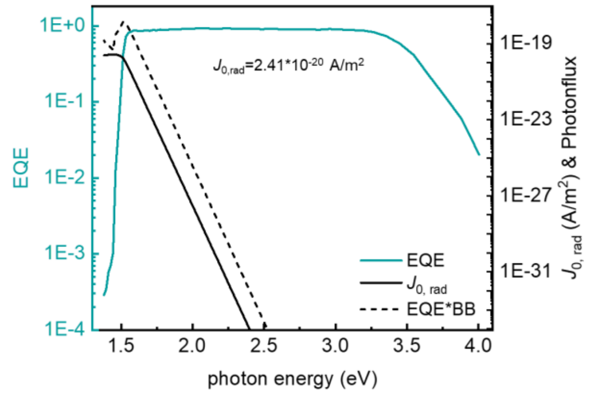

Supplementary Fig. 43. External quantum efficiency (EQE) of the pin PSCs and the emitted spectral photon flux calculated when the device is in equilibrium with the black-body (BB) radiation of the environment at 300 K, the $J_{0,rad}$ is calculated to $2.41*10^{20}$ A/m^2.

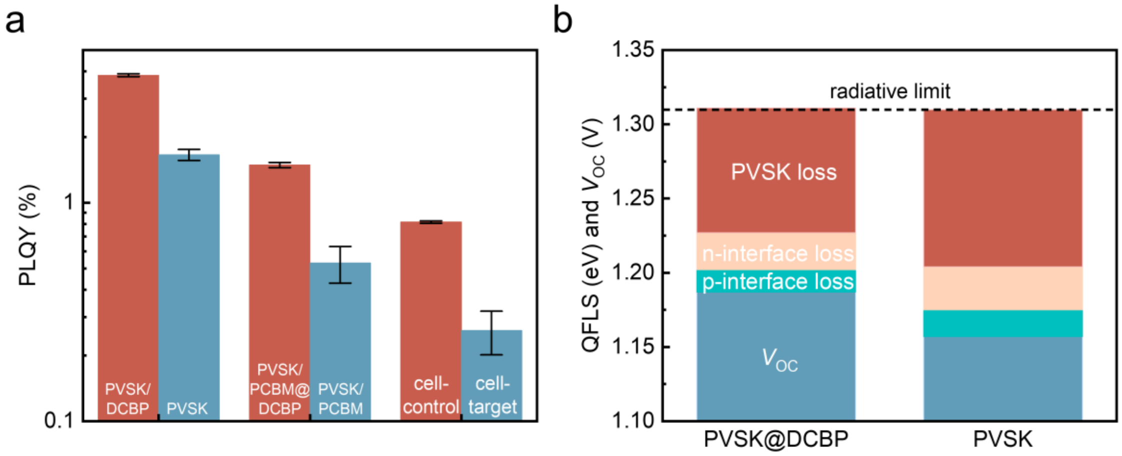

Supplementary Fig. 44. a, Photoluminescence quantum yield (PLQY) diagram for PVSK, PVSK/PCBM half stack, and full cell with/without DCBP. The nearly identical PLQY values observed in PVSK and PVSK/DBCP@PCBM samples suggest the mitigation of interfacial recombination. The error bar representing the standard deviation of three samples. Data are presented as mean values ± s.e.m. **b**, Quasi-fermi level splitting (QFLS) and voltage loss mechanism for the control and DCBP-based samples.

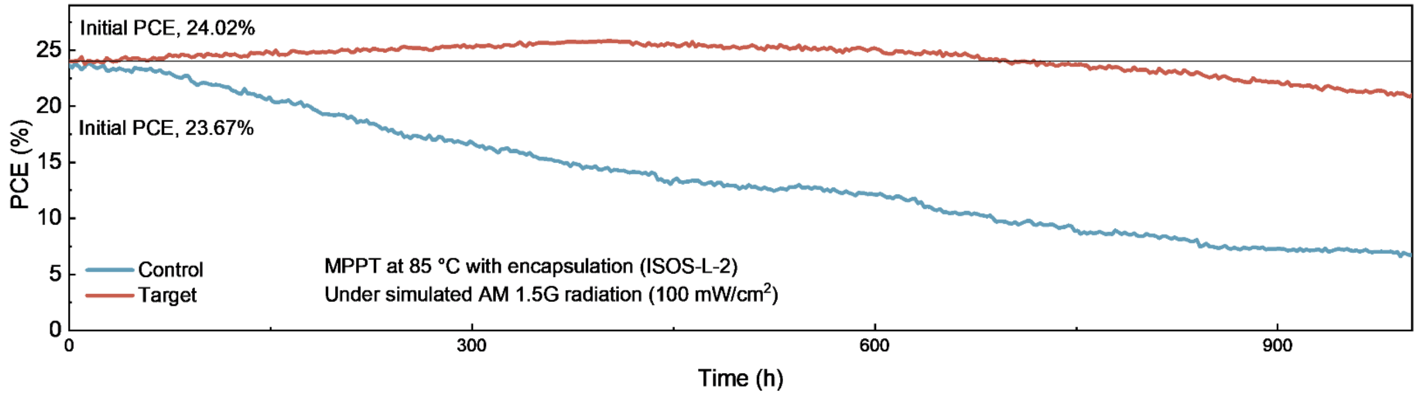

Supplementary Fig. 45. MPPT of the control and target PSCs measured at 85°C under simulated AM1.5G illumination (100 mW/cm^2).

2. Supplementary Tables

Supplementary Table 1. Summary of reported operating stability of inverted PSCs.

Strategy	Electrode	PCE/% (Aperture area/cm^2)	Lifetime/hours (MPP)	T (°C)	Journal/Year
DCBP treated PCBM	Ag	26.03 (0.09)	T_{95} = 2,500	~ 45	This work
MPA-CPA (SAMs)	Ag	25.4 (0.10)	T_{90} = 2,000	~ 45	*Science* (2023, Ref.[1])
H_2O_2 treated NiO_x	Cu	25.5 (0.10)	T_{85} = 1,000	50	*Science* (2023, Ref.[2])
DMAcPA (SAMs)	Ag	25.86 (0.08)	T_{96} = 1,000	~ 25	*Nature* (2023, Ref.[3])
Me-4PACz (SAMs)	Ag	24.5 (0.0585)	T_{90} = 1,200	40 to 50	*Nature Energy* (2023, Ref.[4])
Al_2O_3 (PIC)	Ag	25.6 (0.06)	T_{98} = 1,000	~ 35 to 40	*Science* (2023, Ref.[5])
2PACz/3-MPA (SAMs)	Ag	25.3 (0.049)	T_{98} = 1,075	65	*Nature* (2023, Ref.[6])
(MeO-4PADBC) (SAMs)	Ag	25.6 (0.041)	T_{90} = 1,200	65	*Science* (2023, Ref.[7])
ST treated PVSK	Ag	24.56 (0.049)	T_{96} = 900	~ 35	*Nature Materials* (2023, Ref.[8])
DBSO treated PVSK	Bi/Ag	25.1 (0.09)	T_{97} = 1,800	50 ± 5	*Nature Energy* (2023, Ref.[9])
$DMePDAI_2$ treated PVSK	Au	24.7 (0.12)	T_{90} = 1,000	~ 40	*Science* (2021, Ref.[10])
OLAI treated PVSK	Ag	24.3 (0.07)	T_{90} = 500	~ 40	*Science* (2022, Ref.[11])
$FcTc_2$ treated PVSK	Ag	25.0 (0.08)	T_{90} = 1,500	RT	*Science* (2022, Ref.[12])
Sulfidation treated PVSK	Cu	24.3 (0.09)	T_{90} = 1,000	50 ± 5	*Science* (2022, Ref.[13])

Note:

MPA-CPA: [(2-(4-(bis(4-methoxyphenyl)amino) phenyl)-1-cyanovinyl)phosphonic acid; DMAcPA: (4-(2,7-dibromo-9,9-dimet hylacridin-10(9H)-yl)butyl)phosphonic acid; MeO-4PADBC: (4-(3,11-dimethoxy-7H-dibenzo[c, g]carbazol-7-yl)butyl)phosphonic acid; Me-4PACz: 4-(3,6-dimethyl-9H-carbazol-9-yl)butyl]phosphonic acid; ST: sodium thioglycolate; DBSO: dibutyl sulfoxide; $DMePDAI_2$: N, Ndimethyl-1,3-propane diammonium diiodide; $FcTc_2$: ferrocenyl-bis-thiophene-2-carboxylate; SAM: self-assembled monolayers; PIC: porous insulator contact; RT: Room temperature.

Supplementary Table 2. Summary of reported efficiency of barrier layers modified inverted PSCs.

Strategy	Electrode	PCE/% before modification (Aperture area/cm^2)	PCE/% after modification	Lifetime/ hours (MPP)	T/°C	Journal/Year
DCBP treated PCBM	Ag	23.82 (0.09)	26.03	T_{95} = 2,500	~ 45	This work
YbO_x buffer layer	Cu	- (0.103)	25.2	T_{97} = 1,000	~ 40	*Nature* (2024, Ref.[14])
TTTS treated BCP	Au	19.93 (0.10)	22.59	1,000 (no degradation)	45	*Energy Environ.Sci.* (2021, Ref.[15])
VO_2 barrier layer	Ag	20.96 (0.12)	22.11	T_{90} = 1,000	85	*Adv. Energy. Mater.* (2021, Ref.[16])
BTA treated PCBM	Cu	20.15 (0.09)	19.94	T_{88} = 1,000	RT	*Sci. Adv.* (2020, Ref.[17])
Amorphous ZrN_x diffusion barrier	Cu	22.36 (0.0805)	23.12	T_{88} = 1,500	RT	*Adv. Mater.* (2023, Ref.[18])
C[4]P treated PVSK	Ag	22.5 (0.0625)	23.4	T_{80} = 700	RT	*Adv. Mater.* (2023, Ref.[19])
CI treated PCBM	Au	17.13 (0.04)	21.29	250 (no degradation)	RT	*J. Am. Chem. Soc.* (2021, Ref.[20])
CIL treated PCBM	Ag	15.02 (0.0464)	16.09	T_{80} = 12	RT	*Energy Environ.Sci.* (2016, Ref.[21])

Note:

PCBM: [6,6]-phenyl-C61-butyric acid methyl ester; TTTS: 1,3,5-triazine-2,4,6-trithiol trisodium salt; BCP: bathocuproine; BTA: benzotriazole; DMAcPA: (4-(2,7-dibromo-9,9-dimet hylacridin-10(9H)-yl)butyl)phosphonic acid; CI: cathode interlayer; CIL: chemical inhibition layer; C[4]P: calix[4]pyrrole.

Supplementary Table 3. The binding energy of Ag 3*d* and the kinetic energy of Ag MNN for different Ag samples[22]. PCBM/Ag and PCBM@DCBP/Ag film samples were subjected to 800 hours of continuous simulated AM1.5 illumination aging. The thickness of the Ag electrodes is controlled below 5 nm in order to accurately measure the relationship between the electrode and the under layers.

Sample		Ag 3*d* binding energy (eV)	Ag MNN kinetic energy (eV)
Reference	Ag	368.2	357.9
	Ag_2SO_4	368.3	354.7
	Ag_2O	368.4	350.6
	$AgOOCCF_3$	368.8	355.1
Experiment	PCBM/Ag	368.2	357.9
	PCBM@DCBP/Ag	368.7	354.9

Supplementary Table 4. Calculated valence band (E_{VB}) and conduction band (E_{CB}) from $E_{cut\text{-}off}$, E_F and E_g for the PCBM/Ag and PCBM@DCBP/Ag after continuous simulated AM1.5 illumination aging for 800 hours. The Ag electrodes present were removed using Kapton tape after aging.

Sample	$E_{cut\text{-}off}$ (eV)	$E_{on\text{-}set}$ (eV)	$E_{F\text{-}edge}$ (eV)	E_{VB} (eV)	E_g (eV)	E_{CB} (eV)
PCBM	16.65	1.7	-4.57	-6.27	2.16	-4.11
PCBM@DCBP	16.43	1.74	-4.79	-6.53	2.16	-4.37

Supplementary Table 5. Fitting results of TRPL curves.

Sample	A_1	τ_1 (ns)	A_2	τ_2 (ns)	τ_{avg} (ns)
PVSK/DCBP	0.27	136.6	0.73	689.5	651.7
PVSK	0.34	125.3	0.66	541.5	497.2
PVSK/PCBM/Ag	0.88	88.4	0.12	427.2	222.9
PVSK/PCBM/DCBP/Ag	0.97	27.7	0.03	324.9	106.8

Supplementary Table 6. Photovoltaic parameters of the fresh PSCs based on PCBM or PCBM@DCBP devices. *J-V* curves were measured with a scan rate of 100 mV/s under AM 1.5G illumination.

Devices		J_{SC} (mA/cm^2)	V_{OC} (V)	FF	PCE (%)
Control	Average	24.87 ± 0.24	1.167 ± 0.005	0.801±0.009	23.25 ± 0.41
	Champion	25.12	1.169	0.811	23.82
Target	Average	24.95 ± 0.20	1.172 ± 0.004	0.811±0.006	23.73 ± 0.25
	Champion	25.14	1.178	0.819	24.25

Supplementary Table 7. Photovoltaic parameters of the PSCs based on PCBM or PCBM@DCBP devices after 800 hours of continuous simulated AM1.5 illumination aging. *J-V* curves were measured with a scan rate of 100 mV/s under AM 1.5G illumination.

Devices		J_{SC} (mA/cm^2)	V_{OC} (V)	FF	PCE (%)
Control	Average	21.96 ± 1.42	1.117 ± 0.020	0.723 ± 0.029	17.76 ± 1.50
	Champion	23.83	1.153	0.773	20.52
Target	Average	25.73 ± 0.21	1.176 ± 0.004	0.834 ± 0.004	25.25 ± 0.34
	Champion	26.11	1.184	0.842	26.03

Supplementary Table 8. PLQY and QFLS results of PVSK film, PVSK/PCBM half stack, and full cell with/without DCBP after 800 hours of continuous simulated AM 1.5G illumination aging. Data are presented as mean values ± s.e.m for three samples.

Sample	PLQY (%)		QFLS (eV)	V_{OC} (V)
	Value	Mean values		
PVSK	1.64<br>1.76<br>1.57	1.66 ± 0.096	1.205	
PVSK/DCBP	3.82<br>3.90<br>3.79	3.84 ± 0.057	1.227	
PVSK/PCBM	0.505<br>0.635<br>0.436	0.530 ± 0.101	1.175	
PVSK/PCBM@DCBP	1.49<br>1.42<br>1.53	1.48 ± 0.056	1.202	
PVSK/DCBP/PCBM	1.45<br>1.53<br>1.49	1.49 ± 0.040	1.202	
cell-control	0.325<br>0.232<br>0.216	0.260 ± 0.059	1.157	1.151
cell-target	0.810<br>0.873<br>0.766	0.820 ± 0.054	1.187	1.184

3. Supplementary References

1. Zhang S, et al. Minimizing buried interfacial defects for efficient inverted perovskite solar cells. *Science* **380**, 404-409 (2023).

2. Yu S, et al. Homogenized NiO_x nanoparticles for improved hole transport in inverted perovskite solar cells. *Science* **382**, 1399-1404 (2023).

3. Tan Q, et al. Inverted perovskite solar cells using dimethylacridine-based dopants. *Nature* **620**, 545–551 (2023).

4. Zheng XP, et al. Co-deposition of hole-selective contact and absorber for improving the processability of perovskite solar cells. *Nat. Energy* **8**, 462-472 (2023).

5. Peng W, et al. Reducing nonradiative recombination in perovskite solar cells with a porous insulator contact. *Science* **379**, 683-690 (2023).

6. Park SM, et al. Low-loss contacts on textured substrates for inverted perovskite solar cells. *Nature* **624**, 289-294 (2023).

7. Li Z, et al. Stabilized hole-selective layer for high-performance inverted p-i-n perovskite solar cells. *Science* **6668**, 284-289 (2023).

8. Xu J, et al. Anion optimization for bifunctional surface passivation in perovskite solar cells. *Nat. Mater.* **22**, 1507-1514 (2023).

9. Chen R, et al. Reduction of bulk and surface defects in inverted methylammonium- and bromide-free formamidinium perovskite solar cells. *Nat. Energy* **8**, 839-849 (2023).

10. Zhang F, et al. Metastable Dion-Jacobson 2D structure enables efficient and stable perovskite solar cells. *Science* **375**, 71-76 (2022).

11. Azmi R, et al. Damp heat-stable perovskite solar cells with tailored-dimensionality 2D/3D heterojunctions. *Science* **376**, 73-77 (2022).

12. Li Z, et al. Organometallic-functionalized interfaces for highly efficient inverted perovskite solar cells. *Science* **376**, 416-420 (2022).

13. Li XD, et al. Constructing heterojunctions by surface sulfidation for efficient inverted perovskite solar cells. *Science* **375**, 434-437 (2022).

14. Chen P, et al. Multifunctional ytterbium oxide buffer for perovskite solar cells. *Nature* **625**, 516-522 (2024).

15. Yang JB, et al. Inhibiting metal-inward diffusion-induced degradation through strong chemical coordination toward stable and efficient inverted perovskite solar cells. *Energy Environ. Sci.* **15**, 2154-2163 (2022).

16. Li X, et al. High-efficiency and durable inverted perovskite solar cells with thermally-induced phase-change electron extraction layer. *Adv. Energy Mater.* **11**, (2021).

17. Li XD, et al. Chemical anti-corrosion strategy for stable inverted perovskite solar cells. *Sci. Adv.* **6**, eabd1580 (2020).

18. Xiao MQ, et al. Engineering amorphous-crystallized interface of ZrN_x barriers for stable inverted perovskite solar cells. *Adv. Mater.* **35**, 230168 (2023).

19. Guo HX, et al. Immobilizing surface halide in perovskite solar cells via calix 4 pyrrole. *Adv. Mater.* **35**, (2023).

20. Wang JT, et al. Tuning an electrode work function using organometallic complexes in inverted perovskite solar cells. *J. Am. Chem. Soc.* **143**, 7759-7768 (2021).

21. Back H, et al. Achieving long-term stable perovskite solar cells *via* ion neutralization. *Energy Environ. Sci.* **9**, 1258-1263 (2016).

22. Bin Z, et al. Making silver a stronger n-dopant than cesium via in situ coordination reaction for organic electronics. *Nat. Commun.* **10**, 1-7 (2019).
