# Supplementary material for: Silver coordination-induced n-doping of PCBM for stable and efficient inverted perovskite solar cells
Source: Nat Commun. 2024 Jun 10;15:4922. doi: 10.1038/s41467-024-49395-7 (PMC11164978; doi:10.1038/s41467-024-49395-7)
Supplement: Supplementary file 3 — Reporting Summary [file 41467_2024_49395_MOESM3_ESM.pdf]

nature portfolio

Corresponding author(s): Xiong Li and Zhigang Zang

Solar Cells Reporting Summary

Nature Portfolio wishes to improve the reproducibility of the work that we publish. This form is intended for publication with all accepted papers reporting the characterization of photovoltaic devices and provides structure for consistency and transparency in reporting. Some list items might not apply to an individual manuscript, but all fields must be completed for clarity.

For further information on Nature Research policies, including our data availability policy, see Authors & Referees.

Experimental design

Please check the following details are reported in the manuscript, and provide a brief description or explanation where applicable.

1. Dimensions

Area of the tested solar cells

☒ Yes
☐ No

0.14 cm2 and 1.1 cm2 for small and large-area devices respectively, as described in the Methods.

Explain why this information is not reported/not relevant.

Method used to determine the device area

☒ Yes
☐ No

A black metal mask with an area of 0.09 cm2 and 1 cm2 are used to define the active area of small cell and large-area cell, respectively.

Explain why this information is not reported/not relevant.

2. Current-voltage characterization

Current density-voltage (J-V) plots in both forward and backward direction

☒ Yes
☐ No

The manuscript provides current density-voltage (J-V) plots in both forward and backward direction (Fig. 3a-3d).

Voltage scan conditions

☒ Yes
☐ No

Voltage scan conditions are reported in the manuscript and described in the Methods (Characterization of the solar cells).

Explain why this information is not reported/not relevant.

Test environment

☒ Yes
☐ No

The current-voltage characteristics are obtained under ambient temperature and air conditions, as reported in the Methods (Characterization of the solar cells).

Explain why this information is not reported/not relevant.

Protocol for preconditioning of the device before its characterization

☐ Yes
☒ No

Current-voltage characteristics were recorded in accordance with the procedure reported in the manuscript and in the Methods. No specific protocol for preconditioning was applied.

Explain why this information is not reported/not relevant.

Stability of the J-V characteristic

☒ Yes
☐ No

Maximum power point tracking and damp-heat stability tests were conducted, as reported in the manuscript (Fig. 3g and 3h).

Explain why this information is not reported/not relevant.

3. Hysteresis or any other unusual behaviour

Description of the unusual behaviour observed during the characterization

☒ Yes
☐ No

Very low hysteresis was observed upon altering the scan direction is reported in the manuscript (Fig. 3a-3d).

Explain why this information is not reported/not relevant.

Related experimental data

☒ Yes
☐ No

The manuscript provides current density-voltage (J-V) plots in both forward and backward direction with related experimental data provided in the manuscript (Fig. Fig. 3a-3d).

Explain why this information is not reported/not relevant.

4. Efficiency

External quantum efficiency (EQE) or incident photons to current efficiency (IPCE)

☒ Yes
☐ No

IPCE spectra were recorded, as reported in Fig. 3f.

Explain why this information is not reported/not relevant.

April 2023

A comparison between the integrated response under the standard reference spectrum and the response measure under the simulator

☒ Yes ☐ No

The integrated Jsc from IPCE spectra is consistent with the Jsc from JV measurements, as detailed in the manuscript (Fig. 3b and Fig. 3f).

Explain why this information is not reported/not relevant.

For tandem solar cells, the bias illumination and bias voltage used for each subcell

☐ Yes ☒ No

Provide a description of the measurement conditions.

Not applicable as no tandem solar cells are reported in this work.

5. Calibration

Light source and reference cell or sensor used for the characterization

☒ Yes ☐ No

The current density-voltage (J-V) characteristics of the devices were measured in ambient air (the relative humidity was 40-50%) by an AM 1.5G solar simulator equipped with 450 W Xenon lamp (Newport, 2612A) and a Keithley 2400 source meter. Light intensity was adjusted to AM 1.5G one sun (100 mW/cm2) with a NIM calibrated standard Si solar cell, as detailed in the Methods (Characterization of the solar cells).

Explain why this information is not reported/not relevant.

Confirmation that the reference cell was calibrated and certified

☒ Yes ☐ No

The Si reference cell was calibrated and certified by Newport Corporation PV Lab, Bozeman, MT, USA, as detailed in the Methods.

Explain why this information is not reported/not relevant.

Calculation of spectral mismatch between the reference cell and the devices under test

☒ Yes ☐ No

The spectral mismatch between our simulator and the AM 1.5 solar source was insignificant as the integrated current densities estimated from the IPCE spectra were in good agreement with the values obtained from the current density-voltage (J-V)curves as detailed in the manuscript. Spectra mismatch factor of 1 was used.

Explain why this information is not reported/not relevant.

6. Mask/aperture

Size of the mask/aperture used during testing

☒ Yes ☐ No

All measurements were conducted using a non-reflective metal mask with an aperture area of 0.09 cm2 for small cells and 1 cm2 for large scale cells to cover part of the active area of the device and avoid stray light capturing by our device, as detailed in the Methods (Characterization of the solar cells).

Explain why this information is not reported/not relevant.

Variation of the measured short-circuit current density with the mask/aperture area

☐ Yes ☒ No

Report the difference in the short-circuit current density values measured with the mask and aperture area.

We haven't measured the cells with apertures of different sizes.

7. Performance certification

Identity of the independent certification laboratory that confirmed the photovoltaic performance

☒ Yes ☐ No

The photovoltaic performance of our devices were certified by Shanghai Institute of Microsystem and Information Technology (SIMIT), Chinese Academy of Sciences.

Explain why this information is not reported/not relevant.

A copy of any certificate(s)

☒ Yes ☐ No

Fig. S25.

Explain why this information is not reported/not relevant.

8. Statistics

Number of solar cells tested

☒ Yes ☐ No

At least 15 devices for each condition were tested, as reported (Fig. 3e).

Explain why this information is not reported/not relevant.

Statistical analysis of the device performance

☒ Yes ☐ No

Histograms of efficiency for the devices are reported (Fig. 3e).

Explain why this information is not reported/not relevant.

9. Long-term stability analysis

Type of analysis, bias conditions and environmental conditions

☒ Yes ☐ No

The long-term stability analysis is detailed in the manuscript with respect to the type of analysis, illumination, bias, and environmental conditions (involving temperature and atmosphere humidity), as well as the exclusion of encapsulation, which is detailed in the manuscript (Fig. 3g and 3h).

Explain why this information is not reported/not relevant.
